# Tumor Tissue Detection using Blood-Oxygen-Level-Dependent Functional MRI based on Independent Component Analysis

**DOI:** 10.1038/s41598-017-18453-0

**Published:** 2018-01-19

**Authors:** Huiyuan Huang, Junfeng Lu, Jinsong Wu, Zhongxiang Ding, Shuda Chen, Lisha Duan, Jianling Cui, Fuyong Chen, Dezhi Kang, Le Qi, Wusi Qiu, Seong-Whan Lee, ShiJun Qiu, Dinggang Shen, Yu-Feng Zang, Han Zhang

**Affiliations:** 1 Center for Cognition and Brain Disorders, Hangzhou Normal University, Hangzhou, Zhejiang 311121 China; 2 School of Psychology, South China Normal University, Guangzhou, 510631 China; 3 Zhejiang Key Laboratory for Research in Assessment of Cognitive Impairments, Hangzhou, Zhejiang 310015 China; 4 Department of Neurosurgery, Huashan Hospital, Fudan University, Shanghai, 200040 China; 5 Department of Radiology, Zhejiang Provincial People’s Hospital, Hangzhou, Zhejiang, 310014 China; 6 Department of Neurosurgery, Zhejiang Provincial People’s Hospital, Hangzhou, Zhejiang, 310014 China; 7 Department of Radiology, The Third Hospital of Hebei Medical University, Shijiazhuang, Hebei, 050051 China; 8 Department of Neurosurgery, No.1 Affiliated Hospital of Fujian Medical University, Fuzhou, Fujian 350000 China; 9 Department of Radiology, Affiliated Hospital of Hangzhou Normal University, Hangzhou, Zhejiang 310015 China; 10 Department of Neurosurgery, Affiliated Hospital of Hangzhou Normal University, Hangzhou, Zhejiang 310015 China; 11 Department of Radiology and BRIC, University of North Carolina at Chapel Hill, Chapel Hill, NC 27599 USA; 12 Department of Brain and Cognitive Engineering, Korea University, Seoul, 02841 Republic of Korea

## Abstract

Accurate delineation of gliomas from the surrounding normal brain areas helps maximize tumor resection and improves outcome. Blood-oxygen-level-dependent (BOLD) functional MRI (fMRI) has been routinely adopted for presurgical mapping of the surrounding functional areas. For completely utilizing such imaging data, here we show the feasibility of using presurgical fMRI for tumor delineation. In particular, we introduce a novel method dedicated to tumor detection based on independent component analysis (ICA) of resting-state fMRI (rs-fMRI) with automatic tumor component identification. Multi-center rs-fMRI data of 32 glioma patients from three centers, plus the additional proof-of-concept data of 28 patients from the fourth center with non-brain musculoskeletal tumors, are fed into individual ICA with different total number of components (TNCs). The best-fitted tumor-related components derived from the optimized TNCs setting are automatically determined based on a new template-matching algorithm. The success rates are 100%, 100% and 93.75% for glioma tissue detection for the three centers, respectively, and 85.19% for musculoskeletal tumor detection. We propose that the high success rate could come from the previously overlooked ability of BOLD rs-fMRI in characterizing the abnormal vascularization, vasomotion and perfusion caused by tumors. Our findings suggest an additional usage of the rs-fMRI for comprehensive presurgical assessment.

## Introduction

Glioma is one of the most common but lethal brain tumors. Multimodal neuroimaging-based comprehensive presurgical assessment of the position and the physiological characteristic of the gliomas is essential for radiologists and neurosurgeons^1–3^. Accurate delineation of tumor tissue from the surrounding healthy brain tissues (e.g., eloquent functional areas) helps maximize tumor resection while minimizing potential neurological deficits and thus increases the survival time^4–6^. However, due to the infiltrative, diffusive and migratory characteristics of the gliomas, accurate detection and comprehensive assessment of the glioma entity still poses a great challenge^7,8^.

With high spatial resolution and good tissue contrast, magnetic resonance imaging (MRI) becomes the most commonly used presurgical imaging tool for localization and evaluation of the tumor entity. Conventional MRI techniques, such as the contrast-enhanced T1-weighted, T2-weighted, and T2-weighted fluid-attenuated inversion recovery (FLAIR) MRI, have provided the gold standard for delineating brain tumors from various view angles according to their respective imaging characteristics^7,9^. However, the glioma cells, especially the diffuse lower-grade gliomas, have doubtlessly invaded beyond the T1 contrast-enhanced abnormalities into the normal appearing brain tissue^7,8^, while the T2 or T2-FLAIR imaging are still struggling to differentiate true tumor infiltration from the edema area. Additional physiological measurements, such as from dynamic contrast-enhanced perfusion MRI^10–12^ or dynamic susceptibility contrast-weighted perfusion MRI^13–15^, are thus helpful to provide complementary information for preoperative tumor evaluation and identification^16^. However, the contrast used in the perfusion MRI usually associates with the risk and side effects caused by the contrast agent used. Other imaging modalities, such as magnetic resonance spectroscopy (MRS), diffusion tensor imaging (DTI)/diffusion-weighted images (DWI), and arterial spin labeling (ASL)^17–22^ are non-invasive and free-of-contrast-agent and can also provide tumor morphological and physiological information; but this also introduces additional cost to patients and valuable scanning time.

Blood oxygen level-dependent (BOLD) functional MRI (fMRI) is another useful tool for presurgical planning, as it characterizes neural activity-related changes in the concentrations of oxygenated and deoxygenated hemoglobin concentrations, as well as the fluctuations of the cerebral blood fluid and volume (CBF/CBV). This imaging modality has been widely used in clinical practice for glioma patients, but traditionally contributed only to mapping the surrounding eloquent functional areas (e.g., sensorimotor, visual and language areas) where the resection should be avoided to minimize the risk of irreversible post-operative functional sequelae or neurological deficits^23–25^. Only a few studies have noticed the potential role of BOLD fMRI in tumor entity identification and characterization^26,27^. Compared to the conventional contrast-enhanced imaging, BOLD fMRI is non-invasive, free-of-contrast-agent, and sensitive to abnormal vascularization and perfusion caused by the tumor. Moreover, it provides functional information for time series analysis, allowing more data mining options. In this paper, we sought to use BOLD fMRI and time series analysis to determine potential tumor affected regions, which can provide additional complementary information on tumor localization and other characteristics for better presurgical planning to achieve maximized resection.

Previous BOLD fMRI studies have attempted to use resting-state fMRI (rs-fMRI) to delineate brain tumors from surrounding normal brain tissues by using the co-activity of the “tumor-related” BOLD signals. For example, Feldman *et al*.^26^ demonstrated that the brain tumor could be delineated by the co-activity of both task-based fMRI and rs-fMRI with a seed-based strategy by putting the seed region in the center of the tumors. This study demonstrates that brain tumor may have *unique* BOLD fMRI signals compared with those of other normal brain tissues. Based on rs-fMRI, another recent study conducted by Chamberland *et al*.^27^ found that there were temporal correlations among the BOLD signals inside the tumor by also placing a seed region inside an astrocytoma tumor. However, the segmented tumor was only used for localizing the surrounding white matter fibers. There are several limitations still unsolved. First, there is no *quantitative* evaluation or systematic comparison/validation of the rs-fMRI-based tumor detection. Second, both of the two previous studies only investigated the feasibility using the data with limited tumor types and a small sample size; the feasibility of rs-fMRI-based tumor detection needs more data for evaluation of its *consistency* and *robustness*. Third, the seed-based approach they used has a limitation of unconstraint seed region placement; with different locations of the seeds, the correlation result could be different. It can be challenging to select the *“best”* seed region for different patients with heterogeneous tumors^28,29^.

To solve the drawbacks of seed-based correlation, we proposed to use independent component analysis (ICA), another commonly used method in the rs-fMRI community, for functional network detection, to identify tumors. As a fully data-driven method, ICA does not require any predefined seed region. ICA can also separate noise and artifacts effectively, making the tumor detection robust to imaging noise. In addition, ICA can simultaneously locate multiple functional networks for presurgical functional mapping based on the same rs-fMRI data^30,31^, which is at *no additional cost* for tumor localization. With ICA, rs-fMRI data can be decomposed into multiple spatially independent components based on the spatiotemporal characteristics of the BOLD signals^32,33^. Some of the components have comparable spatial patterns to the task activations using the task-based fMRI; these components are thus believed to support certain function(s) and often called resting-state networks (RSNs) or intrinsic connectivity networks (ICNs)^32,34^. However, in previous ICA applications, other components that locate beyond the canonical RSN/ICN locations were often treated as physiological, instrumental or experimental noises and discarded from further analysis^35^. Different from the normal brain tissues, cerebral tumors can be regarded as a “mix” of abnormal cellular and vascular elements and are usually hypervascularized and desperate for oxygen^26,36,37^. Such histological and metabolic differences between tumor and normal brain tissues may cause changes (often increased) in CBF/CBV, and/or abnormal vascularization, vasomotion or auto-regulation of blood vessels in the tumor-affected regions^38^, which could be sensitively detected by BOLD rs-fMRI. Therefore, we hypothesize that brain tumor has distinct BOLD signals compared to other surrounding normal tissues, and that ICA, as a sensitive blind source separation method, may be able to identify such a “tumor-related” component due to the distinct spatiotemporal BOLD characteristics in the tumor region. However, to our best knowledge, there is no tumor rs-fMRI study reporting such a specific type of components using ICA, which could be a new rs-fMRI ICA application to the clinical field.

In this study, we demonstrated such an additional usage of the preoperatively acquired rs-fMRI by introducing ICA into tumor tissue detection. We developed a largely automated tumor-related component identification algorithm with only minimal human intervention to prevent bias or subjectivity in the conventional manual tumor labeling. This method may serve as a novel, non-invasive, free-of-contrast-agent technique for tumor tissue functional delineation towards comprehensive presurgical assessment. Of note, such an implementation can be easily applied to task-based fMRI, a currently dominant image modality for presurgical functional mapping, because the tumor extraction in our method does not rely on the task-evoked neuronal activity. Our method can also be used to detect other lesions beyond brain tumors. These potentialities could make this new technique broadly interested and widely applicable in future clinical practice.

The contribution of our works is threefold. *First*, this is the first report utilizing BOLD rs-fMRI and ICA to delineate both central nervous system (CNS) and non-CNS tumors preoperatively. Our method allows a non-invasive, free-of-contrast-agent, and abnormal vascularization/perfusion-sensitive tumor delineation. Moreover, it provides more comprehensive and biologically meaningful information by using time series analysis (ICA on the 4D rs-fMRI data), which could better help presurgical planning towards maximized resection. *Second*, this is the first study which demonstrates the feasibility that a tumor-related component can be identified by using ICA successfully, robustly, and reliably, with a template-matching-based, largely automatic component identification method. Such a good performance of ICA is rooted in its abilities to reduce noise and to detect systematic, structured spatiotemporal changes from data. *Third*, we showed that a subject-specific total number of components (TNCs) can be automatically determined and optimized for each individual, instead of using a fixed predefined TNCs for all subjects. With this strategy, we can ensure an optimized tumor-related component detection for each subject, minimizing the risk of unnecessary component splitting or fusion that could impede tumor detection.

## Results

Rs-fMRI (see *Imaging Parameters*) and high-resolution structural MRI data (with or without contrast enhancement, depending on tumor pathology) from a total of 32 glioma patients from 3 different centers (see *Participants*) were included in this study. We intended to include the imaging data with various imaging parameters and different tumor histopathological types to demonstrate the wide application potential or generalizability of our method. The rs-fMRI data was preprocessed (see *Data Preprocessing*) and decomposed by ICA with different settings of TNCs (i.e., model order) (see *Individual Independent Component Analysis*). TNCs is a crucial parameter for ICA which decides how many components will be obtained^39,40^. With different TNCs settings, the resultant tumor-related components may change a lot. Therefore, we sought to develop an algorithm to automatically determine the optimized TNCs to obtain the best tumor component. The best-fitted tumor component was identified by applying our newly proposed algorithm (see *Automatic Tumor-related Component Identification*). For more details, please see *Materials and Methods*.

### Illustrative Case

**Figure 1:**
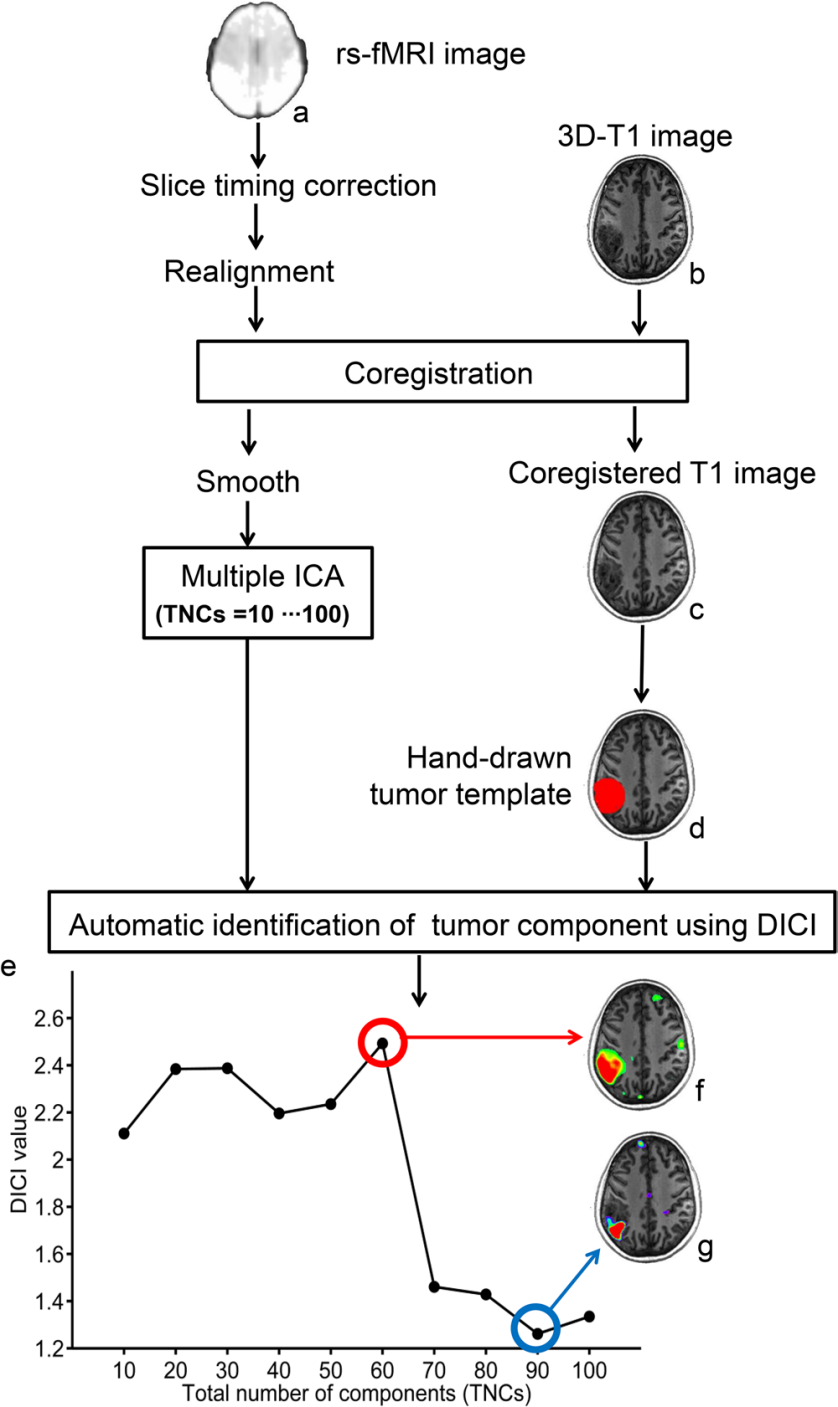
Schematic flow chart and the illustrative result of rs-fMRI-based automatic tumor tissue identification. (**a**) Mean image of a rs-fMRI data; (**b**) raw 3D T1 image; (**c**) T1 image co-registered to the mean rs-fMRI image; (**d**) initial tumor template; (**e**) the curve of the largest DICI values plotted against different TNCs settings; (**f**) final tumor-related component with the largest DICI value across all TNCs settings; and (**g**) a tumor-related component candidate corresponding to the largest DICI value for TNCs = 90 (but not the optimal TNCs).

An exemplary result as well as the data processing procedures are shown in Fig. 1. The patient P #2, randomly selected from the data from Center 1, is a 49-year-old female with astrocytoma (World Health Organization [WHO] grade II) in the left parietal lobe. The tumor can be seen from the T1 image with slightly lower T1-weighted intensity (Fig. 1b) and from the mean Echo Planar Imaging (EPI) rs-fMRI data with slightly higher T2* intensity (Fig. 1a). The hand-drawn tumor template with a widely adopted software roughly captures the tumor region as shown in red in Fig. 1d. We adopted an automatic template-matching algorithm named “Discriminability Index-based Component Identification (DICI)” for the *objective*, *accurate* and *unbiased* tumor-related component identification. A DICI report was generated for this participant for tumor component identification (see Supplementary Table S1 for the detailed DICI report of this subject). The curve of DICI values (the largest DICI values of all components) was plotted against TNCs (i.e., model order) to determine the optimized TNCs and the best-fitted tumor-related component (Fig. 1e). In this case, the TNCs of 60 was the optimized TNCs, which corresponded to the peak of the DICI curve. This TNCs was regarded as the best setting that led to the final tumor-related component (Fig. 1f), while other components with lower DICI values corresponded to the suboptimal results with partial tumor tissue coverage (Fig. 1g). Of note, in this work, our aim is to delineate as entire tumor as possible. Therefore, we treated the partial detection as a suboptimal result. In other works, one may focus on specific tumor compartment, such as the active tumor area or the necrosis; our method is thus not suitable.

### Multi-center Glioma Tissue Identification

**Figure 2:**
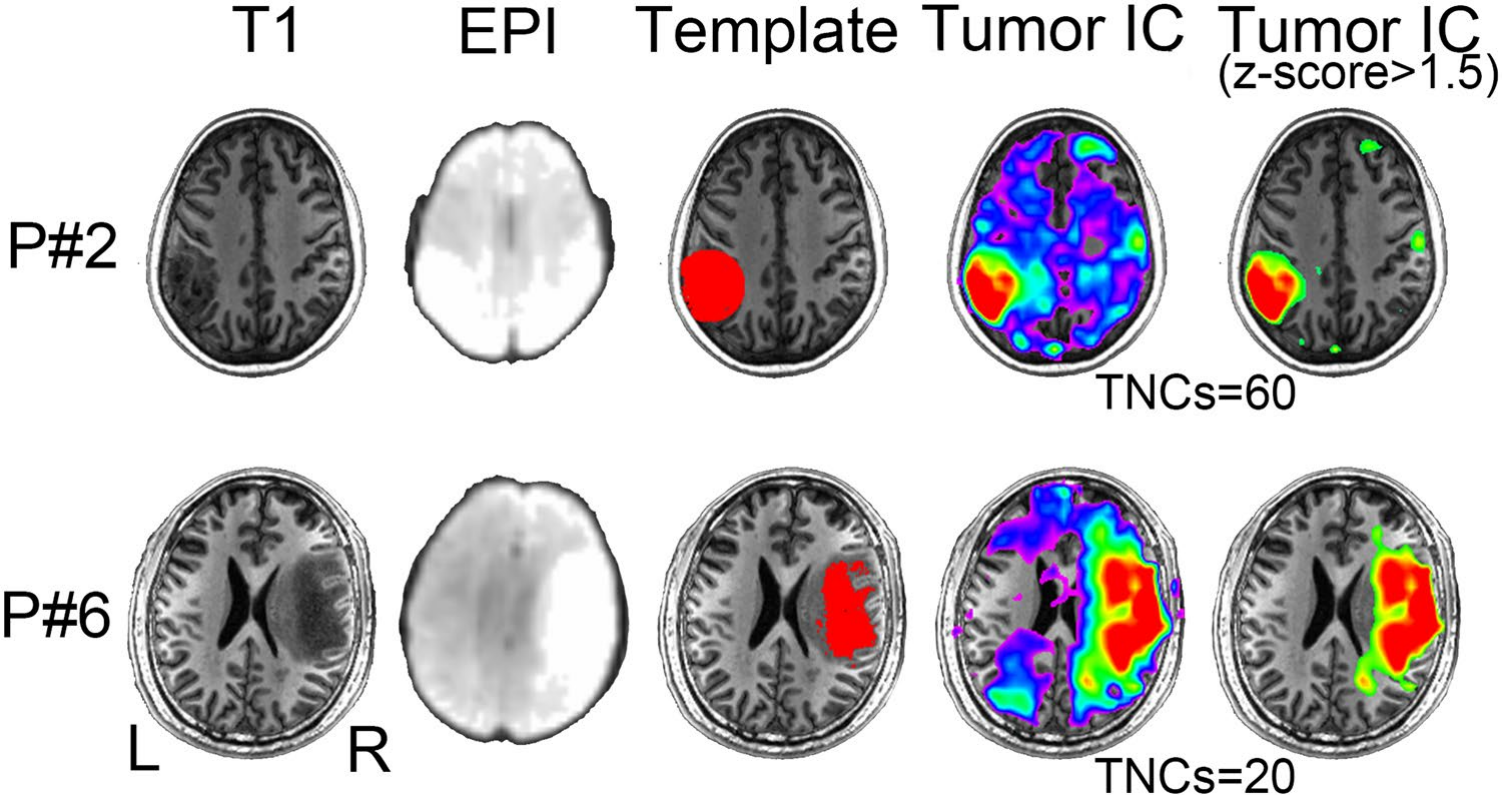
Comparison among 3D-T1 image (1^st^ column), mean EPI image (2^nd^ column), initial tumor template (3^rd^ column), and the final tumor-related components with and without threshold (the last two columns) from two randomly selected patients in Center 1. P*#* denotes the patient ID; TNCs denotes the optimal total number of components as defined by the DICI algorithm; EPI: Echo Planar Imaging; TumorIC: tumor-related component.

**Figure 3:**
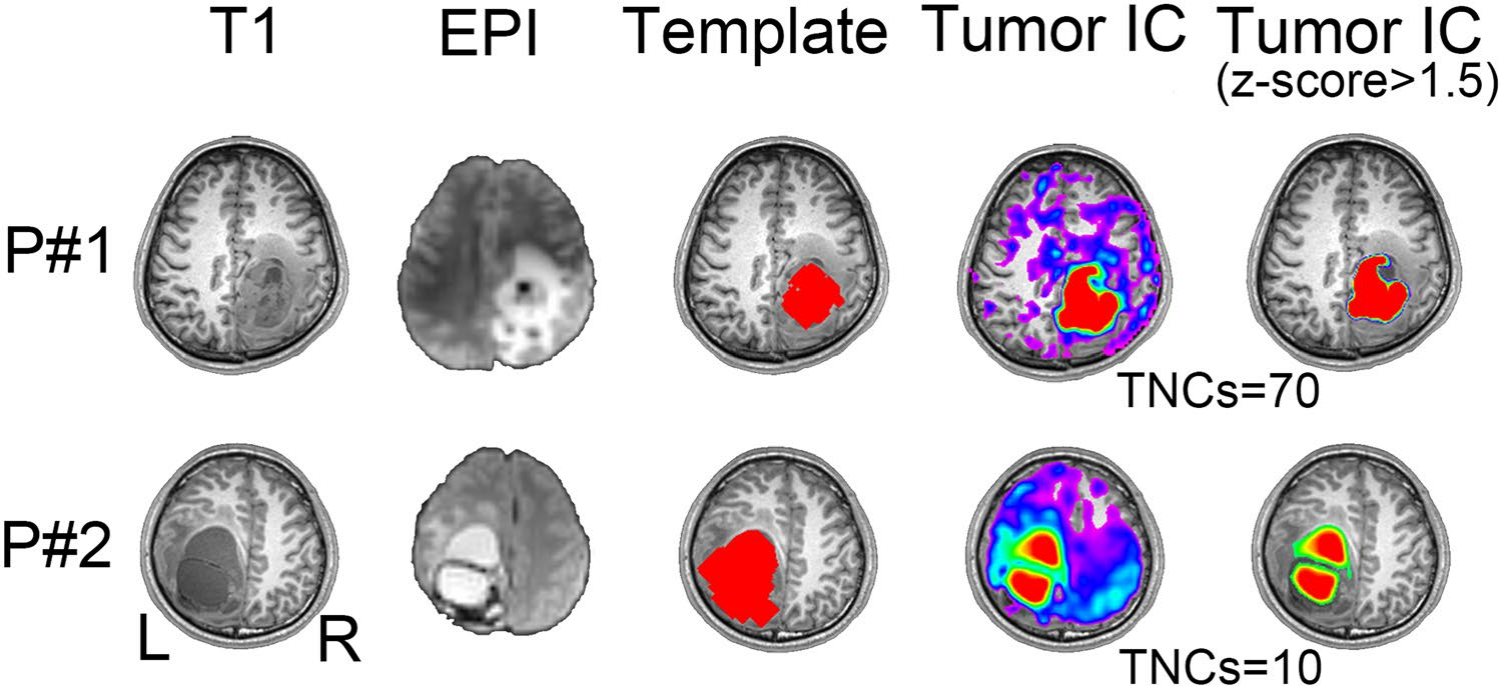
Comparison among 3D-T1 image (1^st^ column), mean EPI image (2^nd^ column), initial tumor template (3^rd^ column), and the final tumor-related components with and without threshold (the last two columns) from two randomly selected patients in Center 2.

**Figure 4:**
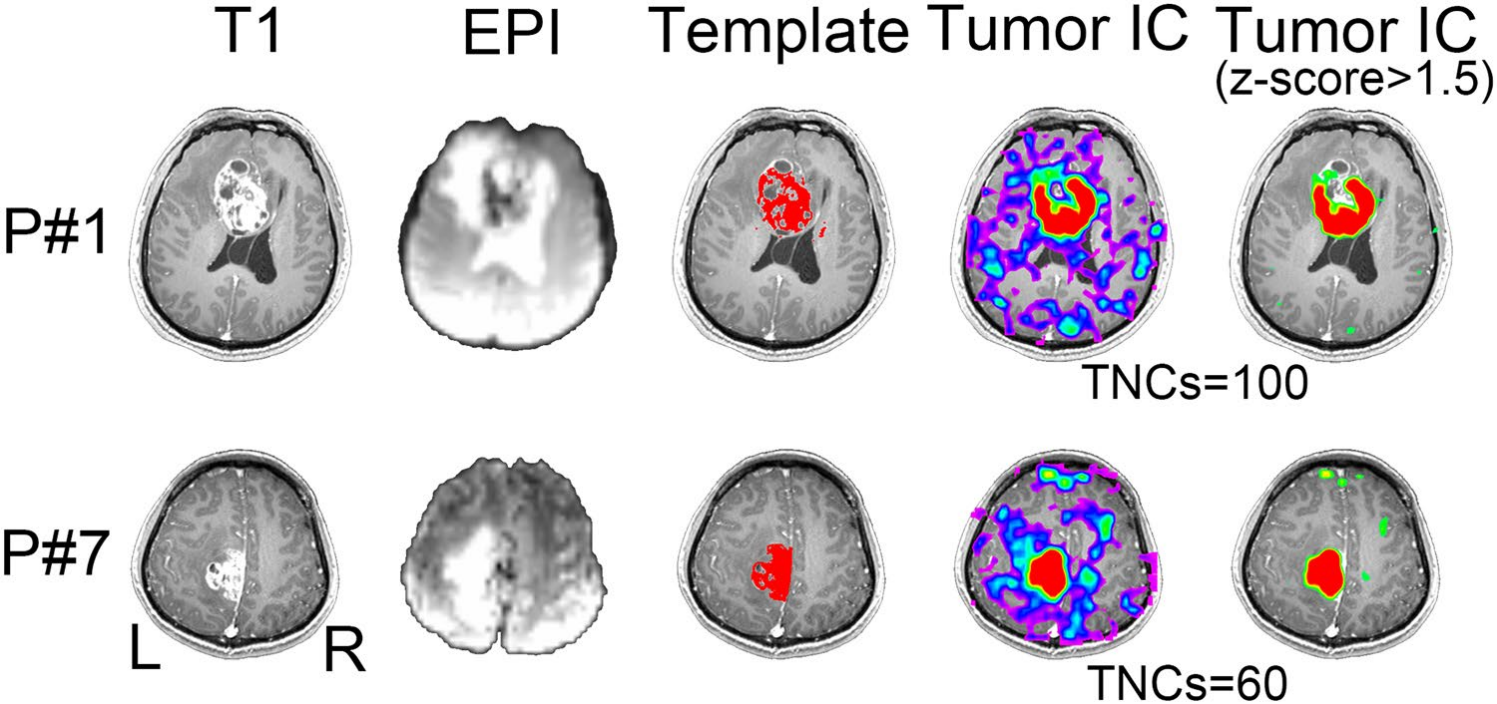
Comparison among 3D-T1 image (1^st^ column), mean EPI image (2^nd^ column), initial tumor template (3^rd^ column), and the final tumor-related components with and without threshold (the last two columns) from two randomly selected patients in Center 3.

We evaluated the feasibility and generalization ability of our new method by using the data from multiple centers, with various imaging parameters and various tumor types (see Supplementary Tables S2–S5 for the data information). Figures 2–4 show the exemplary results of the subjects randomly selected from each of the three centers (Centers 1–3), respectively. Their T1 and mean EPI images were also shown for comprehensive comparisons. Tumor-related components were labeled with warm colors. The results without threshold were also shown for a complete view of the tumor-related components. All these results clearly demonstrated the accuracy of the glioma tissue detection from  these subjects, even with *roughly drawn* tumor templates in some cases. The tumor contrast as shown in the rs-fMRI-based results was more prominent compared with that of the non-enhanced T1 images in several cases.

### Non-brain Tumor Tissue Identification

**Figure 5:**
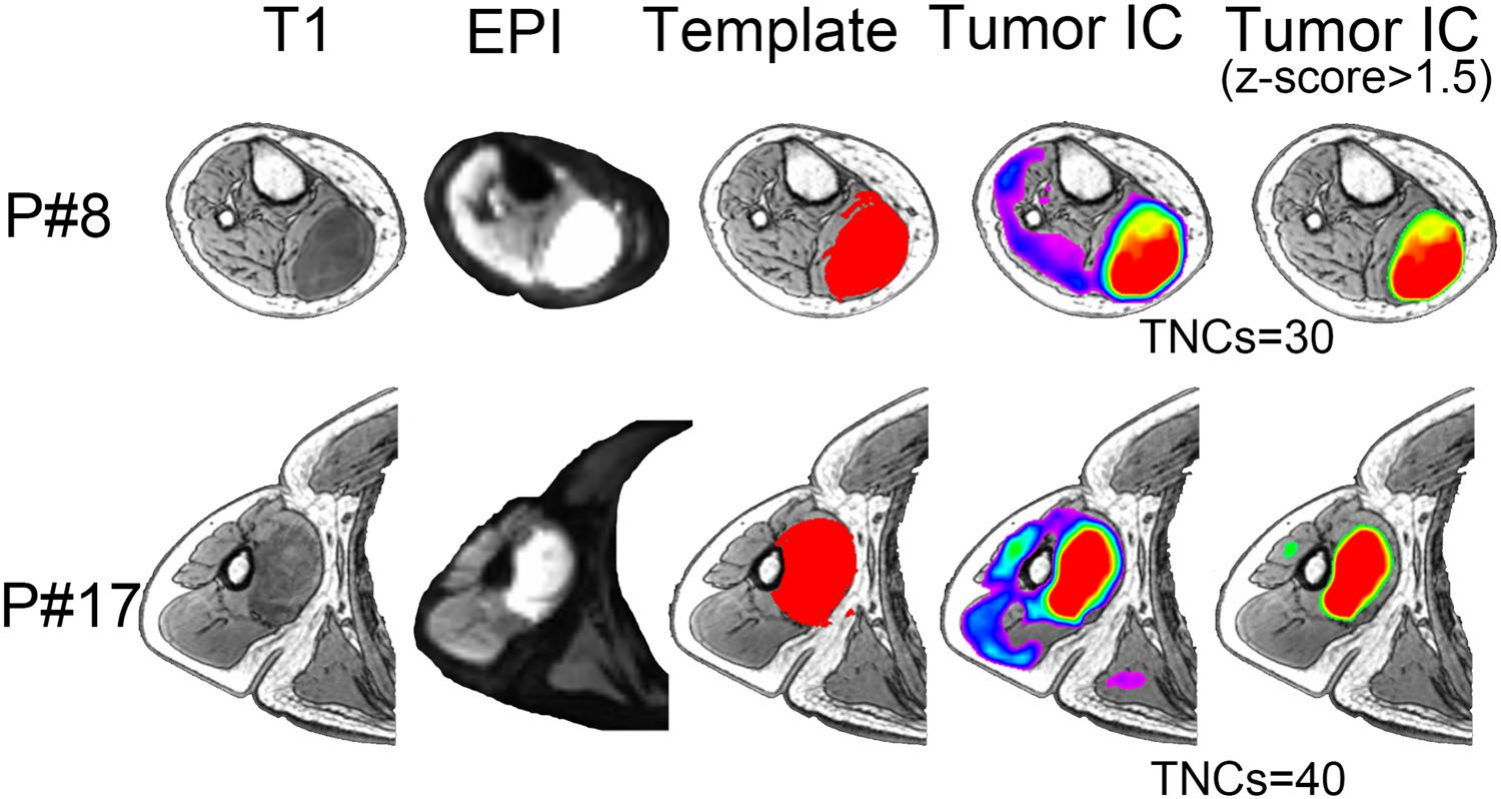
Representative cases for musculoskeletal (MSK) tumor detection. 3D-T1 image (1^st^ column), mean EPI image (2^nd^ column), initial tumor template (3^rd^ column), and finally detected tumor-related components with and without threshold (the last two columns) from two randomly selected patients in Center 4 were compared. The MSK is a non-brain bone and soft-tissue tumor.

The success of rs-fMRI-based brain tumor detection indicates that tumor-related abnormal vascularization and perfusion could result in tumor-specific BOLD signals and lead to the tumor component identified by our method. It is necessary to test this hypothesis using the rs-fMRI data from non-brain tissue with tumors to exclude potential influence by brain-specific processes. Therefore, using the data contributed by Center 4, we further show the performance of our method in detection of bone and soft-tissue tumors for the 28 patients with malignant musculoskeletal (MSK) tumors in the limbs (see Supplementary Table S6). We used such data to demonstrate the potential biological mechanism of the rs-fMRI-based tumor detection, as there is no confounding effect from neural activities. As shown in Fig. 5, the MSK tumors from two randomly selected patients were clearly detected by our method. This result suggests that the neural activity did not contribute to the brain tumor detection.

### Descriptive Statistics of Tumor Identification

The tumor-related components were selected based on the *spatial similarity* to the predefined tumor templates while maintaining *substantial separability* from other components. In other words, we assumed there was *one and only one* component that best matched the tumor area. The analysis of the curves of DICI values against the TNCs settings for ICA helped such identification. Table 1 summarizes the descriptive statistics of each of the first four largest DICI values corresponding to the optimal TNCs setting across all the patients from each center. The averaged 1^st^ largest DICI values were around 2 for all the four centers, which are much higher than the 2^nd^, 3^rd^ and 4^th^ largest DICI values, indicating a good separability of the tumor-related component from other components. On the other hand, the difference between the 2^nd^_,_ 3^rd^ and 4^th^ largest DICI values were not as large as that between the first two largest DICI values, indicating that the component with the 1^st^ largest DICI value was able to represent, if not complete, the majority of the tumor tissue.

**Table 1:** Statistics of DICI values for each center.

Center	1st	2nd	3rd	4th
		1st-2nd	2nd-3rd	3rd-4th
**Center 1**	**2**.**077** (**0**.**793**)	0.910 (0.136)	0.739 (0.121)	0.611 (0.167)
	N.A	**1**.**167** (**0**.**851**)	0.171 (0.122)	0.129 (0.089)
**Center 2**	**1**.**960** (**0**.**630**)	1.006 (0.555)	0.574 (0.330)	0.391 (0.210)
	N.A	**0**.**954** (**0**.**382**)	0.432 (0.305)	0.183 (0.136)
**Center 3**	**1**.**998** (**0**.**760**)	1.142 (0.392)	0.832 (0.457)	0.708 (0.434)
	N.A	**0**.**856** (**0**.**743**)	0.310 (0.286)	0.124 (0.090)
**Center 4**	**2**.**191** (**0**.**575**)	0.730 (0.387)	0.581 (0.348)	0.507 (0.346)
	N.A	**1**.**460** (**0**.**641**)	0.149 (0.153)	0.075 (0.079)

DICI: Discriminability Index-based Component Identification; All values refer to mean (standard deviation). 1^st^ denotes the largest DICI value; 2^nd^ denotes the second largest DICI value; 3^rd^ denotes the third largest DICI value; 4^th^ denotes the fourth largest DICI value; 1^st^ -2^nd^ denotes the difference between the largest DICI value and the second largest DICI value. The others are defined by this analogy. The 1^st^ largest DICI values and the difference between the 1^st^ and the 2^nd^ largest DICI values are highlighted in bold.

Compared with the visual identification results by two experts, the success rate of brain tumor identification was 100% for both Center 1 (8/8) and Center 2 (4/4), and 93.75% (15/16, with one case failed) for Center 3, and the success rate of non-brain tumor identification was 85.71% (24/28) for Center 4 (Table 2). The median, maximum as well as the 25th and 75th percentiles of the optimized TNCs were also listed for the reference of the future studies. Different centers have different mean optimal TNCs. Significant individual variability in the optimal TNCs was observed, which suggests that it is better to detect and use *subject-specific* TNCs setting for each patient instead of using a universal or fixed TNCs setting for all subjects. For one failed case of Center 3, our automatic tumor identification algorithm selected a component that partially covered the tumor region as confirmed by visual evaluation by two experts (Supplementary Figure S1). However, the visual inspection did not find any other component that was better than the automatically suggested component. In all the four failed cases of Center 4, our algorithm failed in the detection of any tumor-related component (see Supplementary Figure S2 for an example of the failed cases). In addition, further visual inspection still could not find any tumor-related component in these four cases. In other words, for these four patients, there was *no* tumor-related component that covered the whole tumor detected by ICA. In all other 24 cases from Center 4 with potential tumor-related components, our algorithm achieved 100% success rate.

**Table 2:** The success rate of tumor tissue identification of each center.

	Center 1	Center 2	Center 3	Center 4
Number of patients	8	4	16	28
Success rate	100%	100%	93.75%	85.71%
Success patient number	(8/8)	(4/4)	(15/16)	(24/28)
Minimal TNCs	20	10	20	10
25th Percentile of TNCs	30	17	50	12
Median TNCs	65	45	60	50
75th Percentile of TNCs	77.5	65	100	80
Maximal TNCs	100	70	100	100

TNCs: total number of components.

### Comparison between ICA and Seed-based Correlation on Tumor Identification

Supplementary Figures S3 and S4 show two examples (P #2 from Center 1 and P #8 from Center 4, with a glioma and an MSK tumor, respectively) comparing the spatial map of the tumor-related component identified by our method (i.e., ICA with DICI algorithm) with the results derived from seed-based correlation as adopted by a few previous studies. The results identified by ICA are better with respect to the spatial coverage of the tumor area. Specifically, the spatial maps of the tumor components identified by ICA almost covered the whole tumor area, indicating good sensitivity and specificity. The seed-based results were *either* much messier (containing excessive noise) with many irrelevant regions included (especially when using a relatively looser threshold) *or* covered only a small part of the tumor entity (when using a more stringent threshold). Moreover, the results of the seed-based analysis were significantly influenced by the placement of the seed region. For example, for the two exemplar cases, although the two seeds were both placed near the center of the tumor entity (but differed a little in the specific location), the results varied prominently.

### Robustness of the DICI Algorithm

Clinical application of our method requires a comprehensive assessment of its robustness. Supplementary Tables S7–S11 show the DICI reports with different thresholds (*z* > 0.5, 1.5, 2, 2.5 and 3) used in the DICI calculation when binarizing the ICA-derived components from a randomly selected patient (P #2 from Ceter 1). The example shows that different thresholds did not affect the final tumor component identification result. No matter which threshold was chosen from *z* = 0.5 to *z* = 3, the component with the largest DICI value and the optimized TNCs remained the same.

## Discussion

In this study, we utilized presurgical BOLD rs-fMRI to delineate tumor tissue in a largely automatic manner. ICA was adopted to decompose individual rs-fMRI data into multiple components. Then, automatic template-matching-based individualized tumor component identification, namely DICI, was conducted to detect tumor-related component with a high success rate (100% success rate for two centers and 93.75% for another center for glioma detection, and 85.71% for the non-brain tumor detection). Tumors with various size, pathological characteristics and locations seem not to hinder the tumor detection. The DICI algorithm was proven robust to the threshold applied for binarizing the components and could tolerate imperfact predefined tumor template. We found large individual variability in the optimized TNCs setting. This is the first time that a subject-specific TNCs setting is calculated explicitly based on the similarity between ICA decomposition results and a specific template of the component-of-interest. For tumor detection, we thus suggest that, instead of using a universal and fixed TNCs setting for all subjects, one needs to identify the optimized TNCs for the best output (specifically, the “tumor-related component detection” in the current study, or the “functional network-related component” in other studies). Finally, the comparison between the performances of our method with that using seed-based correlation further demonstrates the advantage of ICA with automatic component identification. Compared to previous intensity-based tumor delineation techniques using structural imaging with or without contrast enhancement, our method has its specific advantages from *both* modality *and* analysis method viewpoints.

### Advantages of BOLD Rs-fMRI on Tumor Detection

We first analyze the modality advantage for our method. The conventional-MRI-based tumor segmentation methods are primarily based on the signal intensity difference between the tumor tissue and the surrounding normal tissue^41,42^. Some methods jointly utilize T1-, T2- and T2-FLAIR to segment tumors more thoroughly^43,44^, while others use only contrast-enhanced T1 to delineate active tumor tissues^45^. For these methods, multiple high-resolution imaging sequences are required; however, it is still quite difficult to differentiate normal regions from infiltrative tumor tissue using these imaging modalities. For identification of high-grade gliomas, an additional contrast-enhanced imaging is generally needed, further increasing scanning time and risks due to the invasiveness. BOLD rs-fMRI, however, is non-invasive, free-of-contrast-agent, less demanding and, different from all aforementioned imaging modalities, sensitive to abnormal vascularization and perfusion caused by the tumor. Instead of additionally acquire any perfusion MRI, as a usually adopted imaging modality for presurgical functional mapping (for localizing normal functional areas), rs-fMRI can be easily *re-analyzed* (thus at no additional cost) to localize tumor based on the detection of the co-activity of tumor-specific BOLD signals. Because the BOLD fMRI also reflects CBF and CBV as well as depends on normative neurovascular coupling, it could capture tumor-induced abnormalities such as neovasculature, increased CBF/CBV, and deteriorated cerebrovascular reactivity (CVR), thus providing a promising complementary technique to the conventional MRI techniques for the tumor patients.

Our study is not the earliest observation of abnormal BOLD signal in the tumor region. A few previous studies have demonstrated the potential feasibility of using BOLD fMRI to detect brain tumors based on the abnormal BOLD signals inside the tumor, using seed-based correlation method^26,27^. For example, Chamberland *et al*.^27^ put a seed region inside of a glioma and did “functional segmentation” to the tumor. However, the conclusion was based on *only one* subject and the main goal was not for tumor identification, but for nearby white matter fiber tracking. Similarly, Feldman *et al*.^26^ also put seeds in the center of the tumors and the seed-based correlation highlighted the tumor mass, indicating possible “tumor-related” BOLD signals that could help distinguishing tumor from surrounding normal brain tissues. However, the definition of seed regions can be quite tricky; and the cut-off BOLD signal correlation for tumor *vs.* normal tissue differentiation cannot be heuristically determined. These two studies, however, show that rs-fMRI has many merits compared with the task-based fMRI, which makes rs-fMRI less restrictive and easy-to-implement in the clinical setting for surgical planning. In other applications, rs-fMRI has been suggested to be especially useful for the patients with cognitive dysfunction or physical impairment who have difficulty in task executions^31,39,40,46^; and this imaging technique is more and more routinely used nowadays for comprehensive presurgical functional area mapping or determine the dominant language hemisphere^23,31^. We propose that, the same rs-fMRI data originally acquired for presurgical functional mapping can be “re-analyzed” with our method to perform tumor delineation for additional information of tumor distribution for more comprehensive presurgical planning. Therefore, our method may broaden the applications of rs-fMRI into the presurgical planning by adding tumor information to it for achieving the ultimate goal of neurosurgery, i.e., to achieve maximized tumor resection while avoid from impairing vital neurological functions.

Although this is a proof-of-concept study using fMRI to detect tumor areas, we think that it is necessary to compare our result with the traditional, intensity-based tumor detection using T1 contrast-enhanced imaging. We found differences between the two results, which further demonstrates the unique value of fMRI. Specifically, we did a preliminary comparison between the results of conventional MRI-based tumor extraction (using the method of tumor template generation in this study) and that of the fMRI-ICA-based tumor detection (using our proposed DICI method), which is summarized in Supplementary Figure S5. Specifically, we used an intensity-based automatic volume-of-interest (VOI) drawing algorithm implemented by MRIcroN (http://people.cas.sc.edu/rorden/mricron/index.html) to conduct conventional-MRI-based tumor segmentation. This algorithm is based on a regional growth algorithm. The detection result, or the resultant 3D tumor VOI, was further inspected by two neurosurgeons (L.D. and J.C) to make sure it was similar to the manually delineated result (see the “Template” columns in Figs 2–5). For other previously used but more complex tumor segmentation methods, please see^47–52^ for more information. Among them, some algorithms can detect not only the contrast-enhanced area but also the non-enhanced, necrosis and/or edema areas; however, in common clinical practice, delineation of the contrast-enhanced tumor areas is the most important. Therefore, instead of using these complex methods, we adopted a straightforward intensity-based region growth algorithm for T1-based tumor segmentation. Via comparison, we found that the results of our method (based on rs-fMRI and ICA) captured not only the contrast-enhanced areas but also other highly heterogeneous tumor regions, such as the necrosis regions in the center and a small portion of the peripheral hypo-intensity regions (see the differently colored regions in Figs 2–5). This result indicates that, combined with a hand-drawn tumor template, our method could be able to detect complete tumor regions with a simple algorithm.

Of note, to date, rs-fMRI still acts as a complementary technique to support conventional MRI-based presurgical planning such as tumor segmentation in this study. Our study further provides the advantages of the presurgical rs-fMRI scan, which can make this imaging modality more and more popular for surgical planning. The hypothesis that there are distinct BOLD fluctuations between the voxels inside and outside of the tumor regions can be further supported by our study with more samples and multi-center validations.

### Potential Mechanism of BOLD Rs-fMRI-based Tumor Detection

The mechanism of BOLD rs-fMRI-based tumor detection is less clear and complex. Due to limited imaging techniques, we can only give several speculations on the existence of tumor-related components. The BOLD signal depends on the oxygen extraction fraction, oxygenated and deoxygenated hemoglobin concentration, cerebral blood flow/volume and intact neurovascular reactivity^38,53^. The histological and pathophysiological alterations induced by the tumor may change some or all of the above features, thus producing abnormal BOLD signals within tumor compared to the healthy tissue in the vicinity. For example, the blood supply of tumor is similar to hyperemia, the blood flow and the metabolic rate inside of tumor are much higher than the adjacent normal tissue^54^. The success in non-brain tumor detection (Fig. 5) indicated that the information used for tumor identification could not be neuronal orginated. In addition, in line with the previous studies^26,27^, our result shows that the abnormal BOLD signals are highly similar across intra-tumor voxels and distinct from those outsides of the tumor. Such a tiny difference is not mainly contributed by the spatial difference in T2* intensities (for counterexamples please see similar intensity between tumor and non-tumor tissues in the mean EPI data in Figs 2–4) but rather by the difference in temporal variation. This will be thoroughly investigated in one of our ongoing works investigating the differences in the component associated time courses between malignant and benign tumors. This hypothesis may also need to be validated by using other imaging modalities in future, such as the perfusion imaging.

There are other factors that could affect BOLD-fMRI-based tumor detection. First, the BOLD signals inside of the tumor entity could be fundamentally different since the tumor could be highly heterogeneous. For examples, the necrosis regions could have different BOLD signals from the other parts of the tumor; the neovascularized area in a tumor could have different BOLD signals than the peripheral infiltration area. This indicates that ICA could find out multiple “tumor-related” components providing the total number of components is set to be large. Our DICI-based method can automatically determine the component number and reveal the best solution toward a single “tumor component”. Notwithstanding, we still believe our method can also be generalized to the case of multiple “tumor components” by simply giving suggestions based on the largest two or three DICI values (not only one component is chosen, but multiple components are jointly selected). Second, the abnormal vasculature in the neoplasm (e.g., vascular malformations with inappropriately larger size and thinner muscular coat)^55^ could probably alter normal healthy cerebrovascular reactivity and thus affect the BOLD signal. This on one hand gives rise to the “tumor-specific” BOLD signal but, on the other hand, may affect the reliability of BOLD signal and cause failure in detection of the tumor component (e.g., see the failed case in Center 3). This could also be supported by a relatively larger portion of the failed cases for Center 4 with musculoskeletal tumors because there are *less robust* BOLD signals in bones due to the decreased vasculature density. Taken together, rs-fMRI-based tumor detection can be regarded as a complementary technique, which cannot completely substitute the conventional methods that remain to be the “golden standard” for tumor segmentation.

### Advantages of ICA over Seed-based Correlation in Tumor Detection

Previous studies have suggested that ICA and seed-based correlation are the two powerful methods for functional connectivity analysis. While ICA is generally considered performing better in brain functional network detection and fMRI noise separation, while seed-based correlation is also useful due to its intuitive and simplicity. For tumor detection, ICA with dedicated component identification algorithm that considering model order optimization seems to outperform seed-based correlation method without specific consideration of seed position optimization. As the results in Supplementary Figures S3 and S4 indicated, the seed-based tumor delineation can be strongly affected by either the inherent noise of fMRI data or the placement of the seed regions^56^. In addition, it is difficult to choose an appropriate threshold for the best presentation of the tumor tissue due to the significant noise influence. In contrast, ICA, as a fully data-driven approach, requires no prior knowledge of seed placement; the test-retest reliability of ICA on functional network detection generally stays high^57,58^. This is of particular importance for ICA-based individual-level analysis such as presurgical mapping of the functional boundaries of the brain networks. Indeed, a previous study^59^ has reported that ICA-based presurgical functional mapping was more consistent across multiple sessions than the seed-correlation-derived results, with a false positive cluster detected by seed correlation in a repeated fMRI scan, which could be due to the influence of the imaging noise and the weak noise removal ability of seed-based correlation. We thus suggest using individual ICA with dedicated component identification algorithm such as the DICI algorithm for tumor as well as other functional border delineation in the future practice of presurgical planning. Of note, it might be unfair to compare our method (with dedicated parameter optimization) with a simple seed correlation (with non-optimized seed placement). Provide better noise suppression and optimized seed location searching algorithm (it is difficult though since the searching range could be too large), the seed-based correlation may also produce a satisfactory result for tumor delineation. This is because the central idea is to detect co-varied (temporally synchronized) BOLD signals across different voxels. We, in this paper, aim to provide a more convenient method for those with minimal experience without excessive human intervention.

### More Accurate Tumor Detection by Consideration the TNCs Effect

The correct setting of TNCs (i.e., model order) is crucial for ICA^60,61^. With different TNCs settings the resultant tumor-related components may vary significantly (see Fig. 1f and g). If inappropriate TNCs are used, suboptimal decompositions may be obtained, such as the result in Fig. 1g. The TNCs optimization may only be a *scientific issue* in the basic neuroscience studies, however, in presurgical planning applications, this becomes *critical to life*, because a wrong TNCs will negatively affect the preoperative evaluation and could cause an increased risk of irreversible postoperative functional sequelae. Considering large inter-individual variability, we propose that each individual (e.g. brain tumor patients) has its distinct optimized TNCs (or subject-specific TNCs, see Figs 2–5). In the current study, different TNCs settings (ranging for 10 to 100) combined with an automatic component identification algorithm (DICI) were used to determine the optimal TNCs which corresponds to the *best match* to the tumor entity template. The optimal TNCs for selecting tumor-related component was found to be that of the largest DICI value across different TNCs for each participant. TNCs of ICA was optimized based on the ICA result rather than the data property, thus it is more straightforward and goal-directed.

### Methodological Consideration of the DICI Algorithm

Detection of the tumor was designed to be in data-driven and largely automatic fashion, which resulted in high success rate (100% for the two centers and 93.75% for another center of glioma detection, as well as 85.71% for non-brain tumor detection) compared with visual inspection results. We attribute such a success to the merits of automatic component identification DICI method. First of all, our method only introduces minimal human intervention (by only manually drawing a rough initial tumor template using a simple GUI-based software). This not only reduces the laborious works on tumor delineation but also (ideally) avoids time-consuming and biased components selection based on visual inspection. Even for an expert, the more components to be reviewed, the higher level of the error chance. Second, our algorithm is methodologically superior to other previous automatic template-matching methods such as spatial correlation^23,33^ and goodness-of-fit (GOF)^62,63^. These previous methods are sensitive to large variation and extreme values. In contrast, DICI calculations use the thresholded and binarized maps, thus ease the concern of extreme values. In addition, the DICI method uses a distribution-based *z*-score standardization to parameterize the noise’s and signal’s distributions, which could make the component identification more biologically meaningful^64,65^. Finally, the DICI method well weights the sensitivity and specificity and combines them via a reasonable way that is independent of the researcher’s (or rater’s) criteria^64,65^.

The DICI algorithm was originally proposed as a method for automatic detection of a specific brain functional networks. In that application, we hypothesized there was *one and only one* component that fully represents this functional network; So, the goal was to identify a single component that can achieve *both* best spatial match (with the template) *and* best separability (from other components). Therefore, in the original version of DICI algorithm, we calculate two additional outputs for measuring separability, which is the 2^nd^ largest DICI value, and the DICI difference between the largest and the 2^nd^ largest DICI values. In the application for functional network detection, we found that the component corresponding to the largest DICI value was usually the same component that had the largest DICI difference. However, in current tumor detection study, such a previous rule did not always hold. Although the tumor components suggested by DICI *generally* have good separability, we found that, in only a few cases, it was difficult to find a single component that best represented the tumor. This means that, for some subjects with more than one tumor-related components, we could not simultaneously achieve good separability between the best-matched tumor component and other components. We think that this is because the different spatiotemporal sources within a tumor could make the tumor-related component split. Therefore, in the current study, we changed the component selection criteria based on the DICI algorithm a little by giving higher *priority* to identifying the component with the largest DICI value (indicating the best match) over the identification of the component with the largest DICI difference between the first two DICI values (indicating the best separability).

When also considering the separability of the best-matched component, we need to check the DICI values more comprehensively. We need to consider the possibility that multiple components are related to the tumor. In DICI algorithm, such a case indicates poor separability. Although we hypothesized that only one tumor-related component existed in ICA results, however, if the TNCs is set too large, a tumor component could split into two (or more) components. In the case of tumor component splitting, the best match could not be the best solution. Therefore, during DICI calculation, we also took the components with the second, third, and fourth largest DICI values as the candidate tumor-related components. However, via further visual inspiration, we found that, for *most* of the patients, only the component with the largest DICI value was associated with tumor tissue, the other candidate components were either due to noise or related to other functional networks. In other words, there was generally adequate separability between the component with the largest DICI value and those with other DICI values in most of the cases (Table 1). Thus, we only consider the component with the largest DICI value under a specific TNCs setting as the optimal tumor-related component.

### Robustness of the DICI Algorithm

Similar to other template-matching algorithms, DICI also requires a predefined spatial template of tumor regions for tumor-related component identification. In this study, in order to introduce less human intervention (thus reducing the possibility of bias in tumor identification), we only generated rough tumor templates in a largely automatic way using a convenient GUI-based toolbox. We found that tumor could be still accurately and successfully identified, even though the initial template was not perfectly drawn (see the exemplary cases in Figs 1–5). For example, as shown in Fig. 1, the T1 image of the patient indicates that the glioma is in the left parietal lobe (Fig. 1c). The tumor template generated by MRIcroN using the VOI function (Fig. 1d, for details of VOI generation please see *Materials and Methods*) was not perfect but approximates to a sphere. This is because the tumor region has an hypo-intensity appearance in the T1 image with a weak contrast compared to the surrounding brain tissue. Therefore, the region growth algorithm for generating 3D VOI resulted in a template including other non-tumor tissues. However, the tumor-related component identified by DICI (Fig. 1f) was still good, which was much better compared with the initial template in Fig. 1d and consistent with the tumor region as shown in Fig. 1c. Therefore, we think that this simple template generation method is adequate for DICI-based automatic tumor component identification. The reason of the good tolerance to imperfect initial templates for the DICI algorithm could be significantly different BOLD signals between the voxels inside and those outside of a tumor. Another reason is that, in most of the cases, there tends to be a single component representing the whole tumor due to the possible common source(s) that may contribute to the tumor-specific BOLD signals.

Since the DICI calculation requires applying a threshold to binarize the components generated by ICA, it is important to investigate the robustness of the DICI algorithm to such a threshold. We found that the DICI algorithm was robust to various threshold settings (see Supplementary Tables S7–S11). The initial threshold (*z* > 1 and cluster size > 20 voxels) were empirically selected based on our experience and other previous studies, but this is not a strict parameter since we found that the tumor detection result was not changed (i.e., same component at the same TNCs setting was selected) using other thresholds ranging from *z* > 0.5 to *z* > 3. However, such a threshold cannot be set to be very low (e.g., 0, as it is unable to calculate the sensitivity and specificity) or very high (where there is no voxel survived after applying such a threshold, making sensitivity = 0, specificity = 1 and DICI value = infinite). We thus recommend any threshold between 0.5 and 3 for future studies. In addition to DICI calculation, for result visualization, we recommend a more stringent threshold (e.g., *z* > 1.5, compared to *z* > 1 for DICI calculation) to achieve a clearer visualization of the tumor (see Figs 2–5). In future, a smarter threshold determination can be used for both DICI calculation and result visualization. For example, one may use a relative or “soft” threshold such as the percentage of supra-threshold voxels to all brain voxels. In the certain case with a quite low signal-to-noise ratio (SNR) for the rs-fMRI data, even a low threshold can produce zero sensitivity and cause abnormal (e.g., infinite) DICI value. Although it never happens in any of our cases, we still need to be cautious about certain exteme cases. We recommend checking the data first if spurious DICI is obtained.

### Implications from the Failed Cases

Our algorithm did not achieve 100% success in two out of four centers. For glioma detection using three centers’ data, we had one case failed. This is for one subject from the Center 3, where our automatic identification algorithm selected a component that only covered part of the tumor region as found via following visual inspection by experts (see this case in Supplementary Figure S1). As shown in Supplementary Figure S1, the patient had multiple lesions in the brain, but our algorithm only detected one of them and missed the others (especially missed a contrast-enhanced area). However, by visual inspection, we found that there was no other component representing the missing part of the tumor. The component with second largest DICI did not overlap with the tumor. We also inspected the results with other TNC settings, but still could not find any component encompassing this missing part. From the T1 contrast-enhanced image, two major lesions with different contrasts and textures were found. The first part of the lesion had a similar intensity as that of the white matter; and this lesion was identified as a tumor-related component by our method. The other lesion had an enhanced contrast near the first part of the lesion but there was no component covering it. We speculate that the reason why ICA on rs-fMRI did not detect the second lesion is the deteriorated neurovascular reactivity or destroyed vasculature there. This may lead to largely deteriorated BOLD responses and thus low SNR locally, making the ICA-based tumor detection failed.

For non-brain tumor detection using the data from the Center 4, our algorithm failed in detection in slightly more cases (4 out of 28 patients). As shown by an exemplary failed case in Supplementary Figure S2, the patient had a solid MSK tumor in bone as validated by the experts and it could be clearly delineated by using a T1 image with the intensity-based tumor VOI delineation algorithm for initial tumor template generation. However, based on rs-fMRI, our algorithm failed in the detection of any of the tumor-related component. The algorithm suggested a best-fitted component but it only covered a small portion of the bony tumor entity. After going through all components under all TNC settings, we still could not find any better tumor component. For other three failed cases, we had the same findings. We speculate that such results may be due to the fact that the bone and soft tissue generally have relatively fewer blood vessels than the brain tissue, which may cause weaker BOLD signals and reduced SNR of the rs-fMRI signals in this area, making the BOLD-signal coactivity-based tumor identification difficult. In addition, the peak voxel of the automatically suggested tumor component in Supplementary Figure S2 was outside of the tumor. If such a component is really tumor-related, such a phenomenon could indicate the nearby feeding or draining vessels for this MSK tumor.

### Limitations and Future Works

Besides the interesting findings, several limitations need to be noted. First, the sample size of tumor patients from each center needs to increase to further validate our method before clinical application. It needs to be validated based on more cases with different tumor types, locations, sizes and grades, as well as with different fMRI acquisition parameters. Second, if a patient has multiple tumors in the brain, or one tumor entity consists of more than one BOLD fluctuation patterns, our method might only detect one of them, thus limiting its sensitivity. This issue could make the TNCs-induced ICA result variability more complicated. In future, some automated tumor detection algorithms that can detect more lesions need to be integrated into our method to deal with much more complex cases. Third, our method is to determine the best ICA decompositions based on the result (by matching it with a template); some existing ICA algorithms are also promising as they can directly use the prior spatial template information in the ICA optimization process, thus could guide the decomposition toward a specific goal, instead of only data-driven decompositions^66,67^. Fourth, the DICI algorithm only uses spatial information for tumor detection, but ignores the informative temporal (or frequency) information of BOLD fluctuations, which could be helpful for better tumor component identification^63^. Future studies are required to determine the dominant frequency of different tumors due to their distinct vasculature, blood supplies and metabolic rates and their differences compared to those of the healthy brain tissues^54^. Our recent paper on this purpose indicates that different tumor types may have different frequency characteristics^68^. Such information could be integrated into our framework for more accurate tumor identification in the future. Fifth, the 75^th^ percentile of the optimized TNCs for the Center #3 is 100, the same as the upper limit of the TNCs used. It is possible that a larger TNCs may lead to a better DICI value and thus a different component to be selected. A larger searching range of the TNCs may be used in the future. Finally, although we have demonstrated the feasibility of BOLD-rs-fMRI-based tumor detection, the underlying physiological mechanism is still not clear. Additional evidence could come from other imaging modalities such as positron emission tomography (PET), single-photon emission computed tomography (SPECT) and ASL for validation of our hypothesis. In addition, direct evidence from biological mechanism studies using animals or other techniques such as Doppler ultrasound are also highly required. With higher spatial resolution based on the advanced fMRI sequence (e.g., multiband EPI), a detailed investigation of the highly heterogeneous tumor entity may become possible.

## Conclusions

In conclusion, we demonstrated the feasibility of BOLD rs-fMRI-based automatic tumor tissue identification using individual ICA and a novel component identification method named DICI. Both brain tumor and non-brain tumor detection were successful based on the multicenter datasets. This interesting finding suggests that BOLD rs-fMRI can not only be used for presurgical functional mapping, but also is a promising imaging technique for tumor tissue detection. Our method broadens the application of presurgical rs-fMRI and thus is helpful for making a comprehensive presurgical planning.

## Materials and Methods

### Participants

A total of 32 patients with supratentorial glioma were involved. They were scanned in three imaging centers/hospitals. Here we use “Center 1”, “Center 2”, and “Center 3” to represent the names of the imaging centers and subject ID (e.g., P #1) to protect the confidential information of the patients.

To include as various tumor types as possible, the inclusion criteria were slightly different across the three centers. Eight patients from Center 1 were scanned from August 2014 to March 2015; four patients from Center 2 were scanned since July 2010 (two of them were diagnosed to have gliomas but they did not receive any surgery); 20 patients with WHO grade IV (glioblastoma) were randomly selected from the database of Center 3 scanned between 2014 and 2015.

The exclusion criteria consisted of: (1) patients with contraindication to MRI scanning; and (2) patients with other types of head tumors rather than supratentorial gliomas (e.g., meningioma) as suggested by histopathological examination or as diagnosed by radiologists.

All patients were right-handed as assessed by the Edinburgh handedness inventory. The study had obtained local ethics approval by the ethics committee of Zhejiang Provincial People’s Hospital, the ethics committee of No.1 Affiliated Hospital of Fujian Medical University and the Huashan Institutional Review Board. The research was further approved by the ethics committee of the Center for Cognition and Brain Disorders in Hangzhou Normal University. All participants had given informed consent prior to the scan. The methods were carried out in accordance with the approved guidelines. The statistical summary of the demographic and pathological information of all the involved patients can be found in Supplementary Tables S2–S4.

### Imaging Parameters

The MRI data were acquired from different scanners in different centers with various imaging parameters; but for each center, the imaging parameters were the same for all subjects. The imaging protocols consisted of a T2*-weighted EPI BOLD fMRI, and a 3D T1-weighted high-resolution structural MRI with or without contrast enhancement as suggested by initial diagnosis from the radiologists. Supplementary Table S5 listed the scanner information and main imaging parameters used for each center.

### Data Preprocessing

All of the rs-fMRI data processing was carried out using an open-sourced, GUI-based Matlab toolbox dedicated to presurgical mapping (PreSurgMapp, http://restfmri.net/forum/node/2382)^69^. This toolbox is based on DPARSFA v2.3^70^, REST v1.8^71^, SPM8 (http://www.fil.ion.ucl.ac.uk/spm) and MICA beta 1.22^72^ in MATLAB 2012a (the MathWorks, Inc., Natick, MA). The PreSurgMapp seamlessly combines individual (task-based and resting-state) fMRI data preprocessing and post-processing such as individual-level ICA. Since the software is specifically designed for presurgical mapping, all analyses are conducted in each individual’s *native* space rather than the standard space. Therefore, the result will be less affected by image registration that is commonly used for conventional fMRI processing. The whole data processing pipeline is depicted in Fig. 1, including the following preprocessing steps. (1) The first four rs-fMRI frames were discarded by consideration of machine equilibrium and subjects’ adaptation to scanning; (2) Slice timing correction was applied to correct for within-scan acquisition time difference among slices; (3) Patient’s head motion was corrected using a 6-parameter rigid-body transformation, and those with excessive head motion (>3 mm or >3 degrees) were excluded from further analysis. According to this criterion, four patients from Center 3 were excluded; (4) The 3D-T1 image of each patient was co-registered to the same subject’s mean EPI image; and (5) The images were smoothed using a 6-mm full-width-at-half-maximum isotropic Gaussian kernel.

### Individual Independent Component Analysis

Individual-level ICA on each subject’s rs-fMRI data was adopted using the algorithm implemented by MICA embedded in PreSurgMapp. For more details about MICA, please refer to the algorithm paper^72^. Briefly, we used the “initial value-insensitive” ICA decomposition function in MICA. This is because different initial values can introduce variability to ICA result^73,74^. The way to deal with it is to average the results from multiple ICA decompositions, each of which uses a randomized initial value to taking account the stochastic process in ICA optimization^73,74^. The correspondence of the consistent components across *all* ICA runs can be found using a clustering algorithm proposed by Yang *et al*.^75^ in the RAICAR (Ranking and Averaging Independent Component Analysis by Reproducibility) algorithm and the final outputs are the averaged matched components across *all* ICA runs. Although MICA was originally designed to conduct the group-level ICA for a group of subjects using the “subject-order-independent GICA (SOI-GICA)” algorithm^72^, it can be easily applied to single subject fMRI by conducting individual ICA with a consistent result. This will generate reliable tumor-related components. Therefore, in this study, we did not use the subject concatenation order-independence module but only used the initial value-independent module in the MICA. Specifically, each subject’s rs-fMRI data was fed into a one-stage principal component analysis (PCA)-based dimension reduction. After that, ICA decomposition was conducted with the *Infomax* algorithm^76^. Such an analysis was performed for 25 times, each time with a randomized initial value. The resultant multiple results were then integrated to generate the consistent components for each subject^72,77^.

Of note, the TNCs or the model order selection is crucial for ICA^61,78^. With different TNCs settings, the resultant tumor-related components may vary significantly. To identify the optimal TNCs for each subject, different TNCs settings were used for MICA, ranging from 10 to 100 with 10 as an increment. Individual components were transformed to *z*-maps by subtracting global mean and divided by global standard deviation across all voxels^79^.

### Automatic Tumor-related Component Identification

Multiple ICA runs with different TNCs settings will produce a huge number of components to be screened for the tumor-related components, and visual identification is time-consuming, laborious and prone to making mistakes. Therefore, we adopted an automatic, template-matching-based tumor-related component identification method named “DICI”, which utilizes discriminability index (also known as *d*-prime or *d’*) as a template matching or similarity metrics.

The DICI algorithm requires a predefined tumor spatial template, which can be manually drawn by experts; but it is again laborious and error-prone. In order to reduce human intervention, we proposed in our method to generate a rough tumor template in a largely automatic way using a simple intensity-based region growth algorithm as implemented in a user-friendly GUI-based toolbox. We found that, with such a roughly-drawn (even though not quite perfectly) initial template, the tumor could be successfully detected (see the exemplary cases in *Results*). For further validation, we also manually drew the tumor templates and the results were the same as those based on the roughly drawn templates. Therefore, we believed that this simple method was adequate for following tumor detection.

Specifically, the individual tumor template was generated based on each patient’s T1 image via an automatic VOI drawing function in MRIcroN. We first loaded the co-registered T1 image into MRIcroN and put the crosshair approximately at the center of the tumor entity. We then used ‘Create 3D VOI based on background intensity’ function in MRIcroN to create a tumor 3D VOI centering at the crosshair. The algorithm includes three parameters (*“Radius”*, *“Difference from origin”*, *“Difference from edge”*) that may vary the shape of the VOI. The “Radius” was adjusted to roughly cover the whole tumor. The “Difference from origin” was adjusted to make the VOI roughly cover the region with abnormal T1 intensities while not including too much surrounding normal brain tissue. By adjusting the *“Difference from edge”*, we further polished the edge of the VOI. Finally, the VOI created was saved and visually validated by a radiologist (Z.D.). This is a simple intensity-based region growth algorithm, and a well-trained researcher will take as short as 30 seconds to generate this tumor template without the need of time-consuming manual refinement. This strategy worked in all our cases from all centers.

Discriminability Index (from now on, we call it DICI in the context of Component Identification) was used to measure the spatial similarity between each component and the predefined tumor template. DICI was originally adopted in signal detection theory in the fields of experimental psychology and information theory^64,65,80^, where it is defined by the distance from the center of the signal distribution to that of the noise distribution. It measures to what extent the signal can be separated from noise. Since tumor component identification can also be regarded as a problem of signal (tumor component) vs. noise (other components) judgment and the goal is to find a good separation between the tumor component and other components, DICI is quite natural to be borrowed for our study. To calculate it, each component (*z*-map) was first converted to a binary map by applying a threshold (*z* > 1, cluster size > 20 voxels). Then, according to Table 3, DICI value was calculated by comparing the binary map to the binary tumor template. Both sensitivity and specificity were calculated. The sensitivity is also called hit rate (HR, i.e., the number of correctly identified voxels within a component divided by the total number of voxels in the tumor template); the specificity is one minus false alarm rate (FAR) (the number of mistakenly identified voxels within a component divided by the number of voxels outside the tumor template). Both HR and FAR were transformed to *z*-scores using an inverted cumulative distribution function of a standard normal distribution (mean = 0 and standard deviation = 1). A good matching indicates a high HR and a low FAR; therefore, DICI combines the two rates by using *z*(HR) minus *z*(FAR).

**Table 3:** Calculation of discriminability index for component identification (DICI).

	Voxels labeled “1” in the component (*significant voxels*)	Voxels labeled “0” in the component (*non-significant voxels*)	**DICI** = **z**(**HR**)**-z**(**FAR**)^a^
Voxels labeled “1” in individual tumor template	**Hit** (*true positive*)	**Miss** (*false negative*)	**Hit rate** (**HR**) = hit/voxels labeled “1” in individual tumor template
Voxels labeled “0” in individual tumor template	**False alarm** (*false positive*)	**Correct rejection** (*true negative*)	**False alarm rate** (**FAR**) = false alarm/voxels labeled “0” in individual tumor template

^a^The HR and the FAR was transformed to *z-*scores according to an inverted cumulative distribution function of a standard Gaussian distribution (mean = 0 and standard deviation = 1).

For the ICA result with each TNCs setting, all components were sorted in a descending order based on their DICI values. The component with the *largest* DICI value was selected as a tumor-related component *candidate*. To evaluate potential component splitting, we also recorded the second, third and fourth largest DICIs. Aside from this, the difference in DICI value (ΔDICI) among the largest four DICIs were pair-wisely calculated to assess the *separability* of ICA. Such a procedure was conducted for all TNCs settings, resulting in a set of tumor-related component *candidates*. The DICI values of all the candidates were compared again to identify a single candidate with the largest DICI values of all candidates. This single component was selected as the final tumor-related component, and its corresponding TNCs was defined as the optimal TNCs for tumor detection. Based on our experience on DICI-based brain functional network detection, we assumed that the identified tumor-related component had *both* the highest similarity to the tumor template and adequate separability from the components with the second, third and fourth largest DICI values. This assumption held true for most of the cases (see *Results*), indicating there was only one tumor-related component. For the cases with potentially multiple tumor components, please see *Discussion*.

All the automatically selected tumor-related components were visually validated by two experts (H.H. and H.Z.). For such visual inspection and result presentation, each tumor-related component was overlaid on each subject’ own 3D-T1 image with a threshold of *z* > 1.5 and was further compared with the region(s) with abnormal T1 intensity.

### Non-brain Tumor Tissue Identification

To further investigate potential biological mechanisms of the BOLD rs-fMRI-based tumor detection, we applied our method to another dataset with non-brain tumors. In this dataset, the tumors were not located in the central nervous system. Because the BOLD signals from non-brain regions have no relationship with neuronal activities or glial cells but may reflect the abnormal blood supply and vasculature as the brain tumors, we can thus exclude the potential influence of neuronal activities from the potential mechanisms of BOLD rs-fMRI-based tumor detection. Besides, we can further test the potential wider applications of our method to detect various types of tumors.

The rs-fMRI data was collected from MSK tumor patients in the Center 4. Totally 28 patients were randomly selected from the MSK neuroimaging database in the Center 4, collected between February 2009 to June 2011 with a Siemens 1.5T scanner^68^. See Supplementary Table S5 for detailed imaging parameters and Supplementary Table S6 for demographic and pathological information of the patients involved. The study was approved by the institutional research ethics board in the Third Hospital of Hebei Medical University, and all patients signed a written informed consent before the study was carried out. The MSK tumor-related component was automatically identified based on our method and visually validated by two experts (L.D. and J.C.).

### Descriptive Statistical Analysis

To quantitatively evaluate the performance of our method, for each center, simple descriptive statistics were calculated including the mean and standard deviation of DICI values for the first four largest DICI components across all subjects within each center. The mean and standard deviation of the DICI differences (∆DICI) were also calculated to evaluate separability. The median of the optimal TNCs for each center was also shown. The successful tumor component identification was counted for each center based on whether the automatically identified tumor component was in agreement with two experts’ decision. Of note, the two experts independently viewed all the components and identified the tumor-related component. They reached agreements for all cases including four cases in Center 4 with no tumor-related component identified by both experts. For one case in Center 3 both experts selected a component with partial tumor coverage (see *Results*). The performance of our method was evaluated by the success rate for each center, which was calculated by using the number of successfully identified cases divided by the total number of cases for each center.

### Comparison between ICA and Seed-based Correlation on Tumor Identification

We further compared the performance of ICA-based tumor identification with that based on seed correlation, another widely used method in the resting-state BOLD fMRI community and fMRI-based brain tumor segmentation studies^26,27^. The processing steps for the seed correlation-based tumor delineation were the same as the previous studies^26,27^. Considering the tumor detection result could depend on the seed-region placement, for each patient, we defined two spheric seed ROIs (radius = 6 mm) near the center of the tumor entity based on the best judgment of the authors and conducted seed-based correlation for each seed, separately. The average BOLD signal of each seed region was extracted, and Pearson’s correlations were computed between the seed BOLD time series and those from all other brain voxels. Since the threshold determination is also important to the sensitivity and specificity of seed-correlation-based tumor delineation, different correlation coefficient thresholds (*r* > 0.5, *r* > 0.8) were applied separately to the resultant correlation maps and they were both compared with the best-fitted tumor component derived by ICA based on DICI.

### Robustness of the DICI Algorithm

We evaluated the robustness of the DICI algorithm with respect to roughly (not precisely) drawn initial tumor templates and different component binarization thresholds. We assumed that the DICI algorithm would work well even using a roughly drawn tumor template. For this purpose, we chose a subject for which the intensity-based tumor template generated by MRIcroN was not quite consistent with the manually drawn template. The same tumor component identification method was applied using the two different templates and the results were compared. Since DICI calculation involves sensitivity and specificity calculation, the threshold chosen could be an influencing factor to the DICI values and final tumor identification result. We compared the DICI-based tumor identification results using different binarization thresholds for a randomly selected subject. We adopted different threshold settings (from *z* = 0.5 to *z* = 3 with an increment of 0.5) and conducted the same tumor component identification algorithm to examine whether the finally selected tumor component could be changed due to different thresholds used.

## Electronic supplementary material

Supplementary Information
