# Supplementary material for: Tumor Tissue Detection using Blood-Oxygen-Level-Dependent Functional MRI based on Independent Component Analysis
Source: Sci Rep. 2018 Jan 19;8:1223. doi: 10.1038/s41598-017-18453-0 (PMC5775317; doi:10.1038/s41598-017-18453-0)
Supplement: Supplementary file 1 — Supplementary Information [file 41598_2017_18453_MOESM1_ESM.pdf]

Supplementary Information

Tumor Tissue Detection using Blood-Oxygen-Level-Dependent Functional MRI based on Independent Component Analysis

Huiyuan Huang[1,2,3], Junfeng Lu[4], Jinsong Wu[4], Zhongxiang Ding[5], Shuda Chen[6], Lisha Duan[7], Jianling Cui[7], Fuyong Chen[8], Dezhi Kang[8], Le Qi[9], Wusi Qiu[10], Seong-Whan Lee[11], ShiJun Qiu[12], Dinggang Shen[11,13]*, Yu-Feng Zang[1,3], Han Zhang[1,3,13]*

[1]Center for Cognition and Brain Disorders, Hangzhou Normal University, Hangzhou, Zhejiang 311121, China
[2]School of Psychology, South China Normal University, Guangzhou 510631, China
[3]Zhejiang Key Laboratory for Research in Assessment of Cognitive Impairments, Hangzhou, Zhejiang 310015, China
[4]Department of Neurosurgery, Huashan Hospital, Fudan University, Shanghai, 200040, China
[5]Department of Radiology, Zhejiang Provincial People's Hospital, Hangzhou, Zhejiang 310014, China
[6]Department of Neurosurgery, Zhejiang Provincial People's Hospital, Hangzhou, Zhejiang 310014, China
[7]Department of Radiology, the Third Hospital of Hebei Medical University, Shijiazhuang, Hebei 050051, China
[8]Department of Neurosurgery, No.1 Affiliated Hospital of Fujian Medical University, Fuzhou, Fujian 350000, China
[9]Department of Radiology, Affiliated Hospital of Hangzhou Normal University, Hangzhou, Zhejiang 310015, China
[10]Department of Neurosurgery, Affiliated Hospital of Hangzhou Normal University, Hangzhou, Zhejiang 310015, China
[11]Department of Brain and Cognitive Engineering, Korea University, Seoul 02841, Republic of Korea
[12]Medical Imaging Center, The First Affiliated Hospital of Guangzhou University of Chinese Medicine, Guangzhou, Guangdong, 510405 China
[13]Department of Radiology and BRIC, University of North Carolina at Chapel Hill, NC 27599, USA

* Corresponding authors:
Han Zhang, Ph.D.
Emails: hanzhang@med.unc.edu; hanzhang@hznu.edu.cn
Tels: + 1(919) 808 8965; +86-571-28867717
Dinggang Shen, Ph.D.
Email: dgshen@med.unc.edu
Tel: +1(919) 843 5420

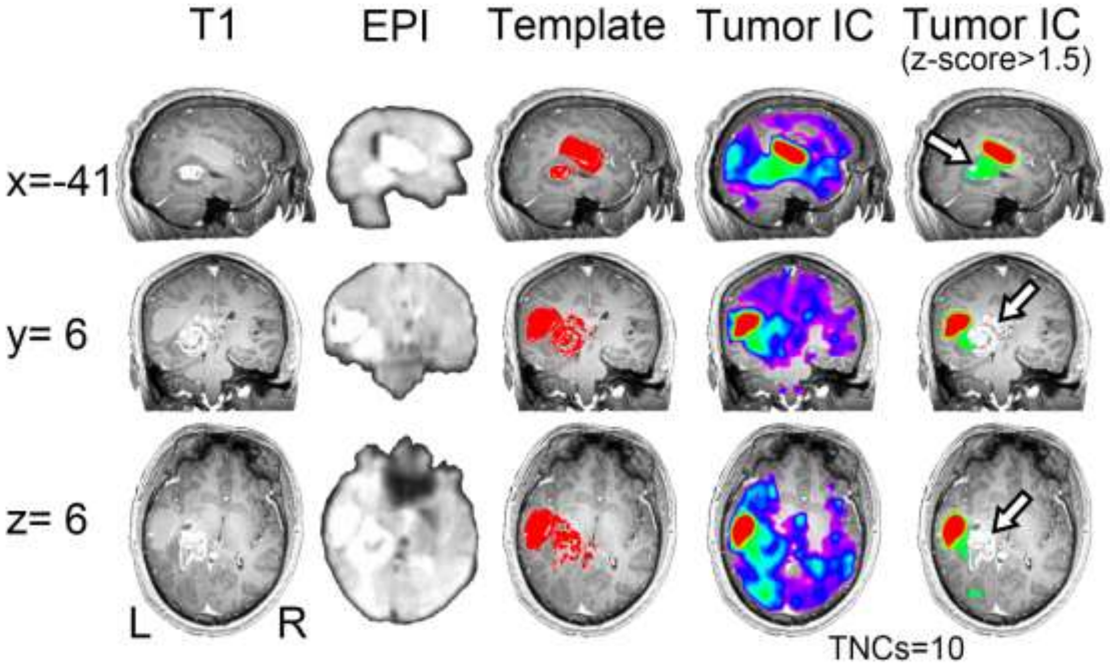

Supplementary Figure S1. A case of glioma identification with a suboptimal result. 3D-T1 image (1st column), mean EPI image (2nd column), initial tumor template (3rd column), and tumor-related components with and without threshold (the last two columns) are shown. TNCs here denotes the optimal total number of components as defined by our DICI algorithm; EPI: Echo Planar Imaging; Tumor IC: tumor-related independent component. Our method identified a part of the tumor region, but did not include the additional contrast-enhanced region (as indicated by arrows). However, if no threshold was applied, the contrast-enhanced region seems to connect with the other tumor area.

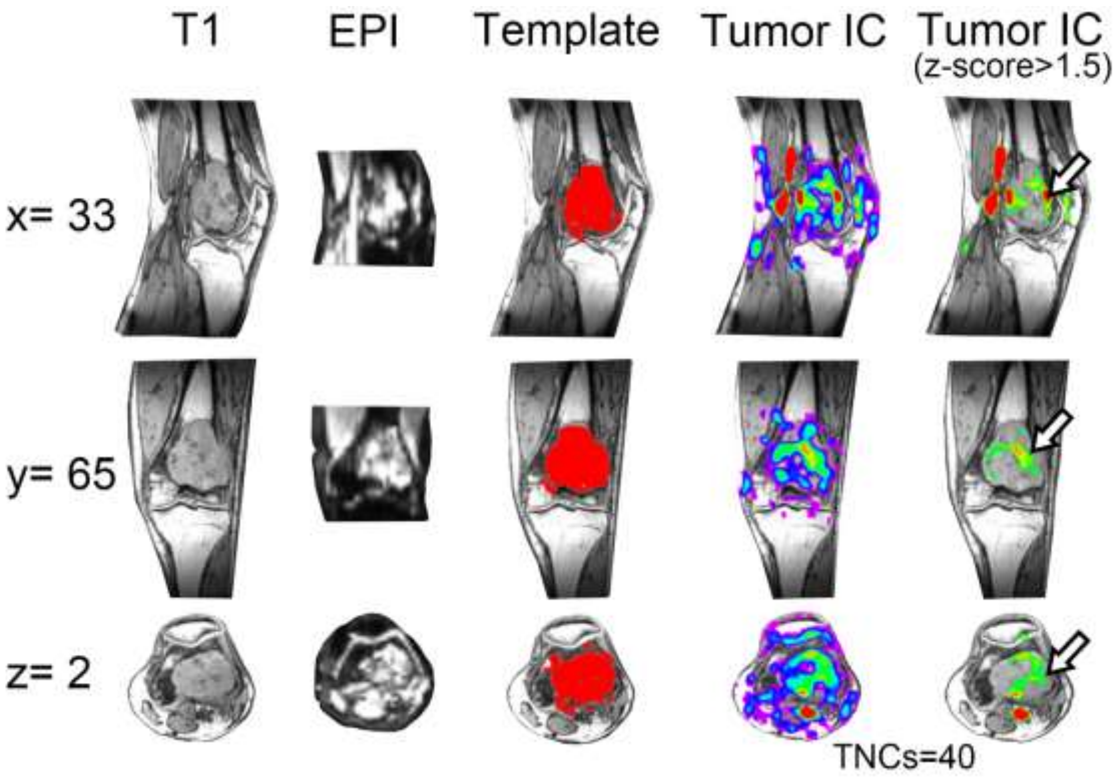

Supplementary Figure S2. A case of non-brain musculoskeletal (MSK) tumor identification with a suboptimal result. 3D-T1 image (1st column), mean EPI image (2nd column), initial tumor template (3rd column), and tumor-related components with and without threshold (the last two columns) were shown. TNCs here denotes the optimal total number of components as defined by our DICI algorithm; EPI: Echo Planar Imaging; Tumor IC: tumor-related independent component. Our method identified a part of the tumor region, but the other part of the tumor region was not identified (as indicated by arrows). The red colored areas in the "Template" column are roughly the whole tumor. Tumor IC has relatively weak signals compared with the peak region that could be outside of the tumor area.

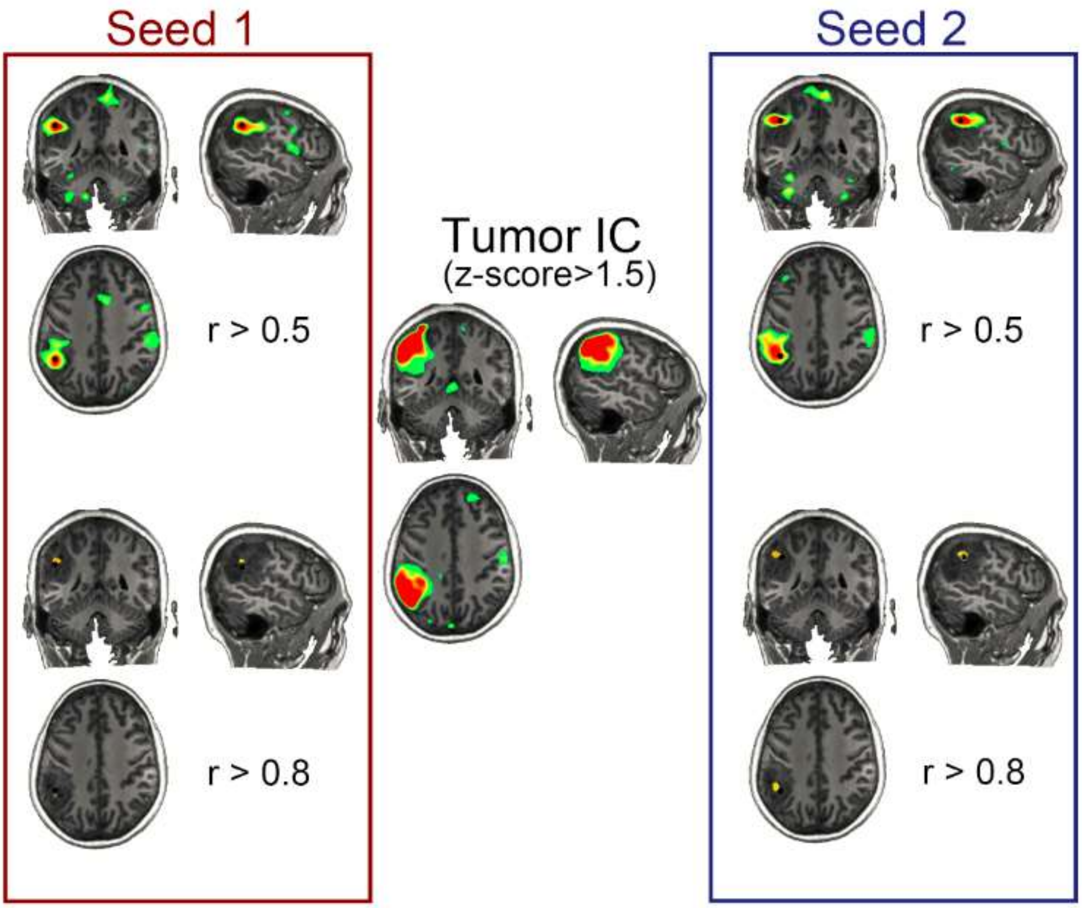

Supplementary Figure S3. Representative case (P #2 from Center 1) showing the spatial map of the ICA-based tumor-related component (in the center) and those from the seed-based correlations using different seeds and different thresholds. This patient had a glioma in the left parietal lobe. Seed 1 and Seed 2 represent the seed correlation-based results with each seed region placed at different voxels in the center of the tumor (as indicated by the black dots); Tumor IC: tumor-related component derived from ICA and identified by our DICI algorithm. Different thresholds (0.5 and 0.8) of the correlation maps were used to show the results.

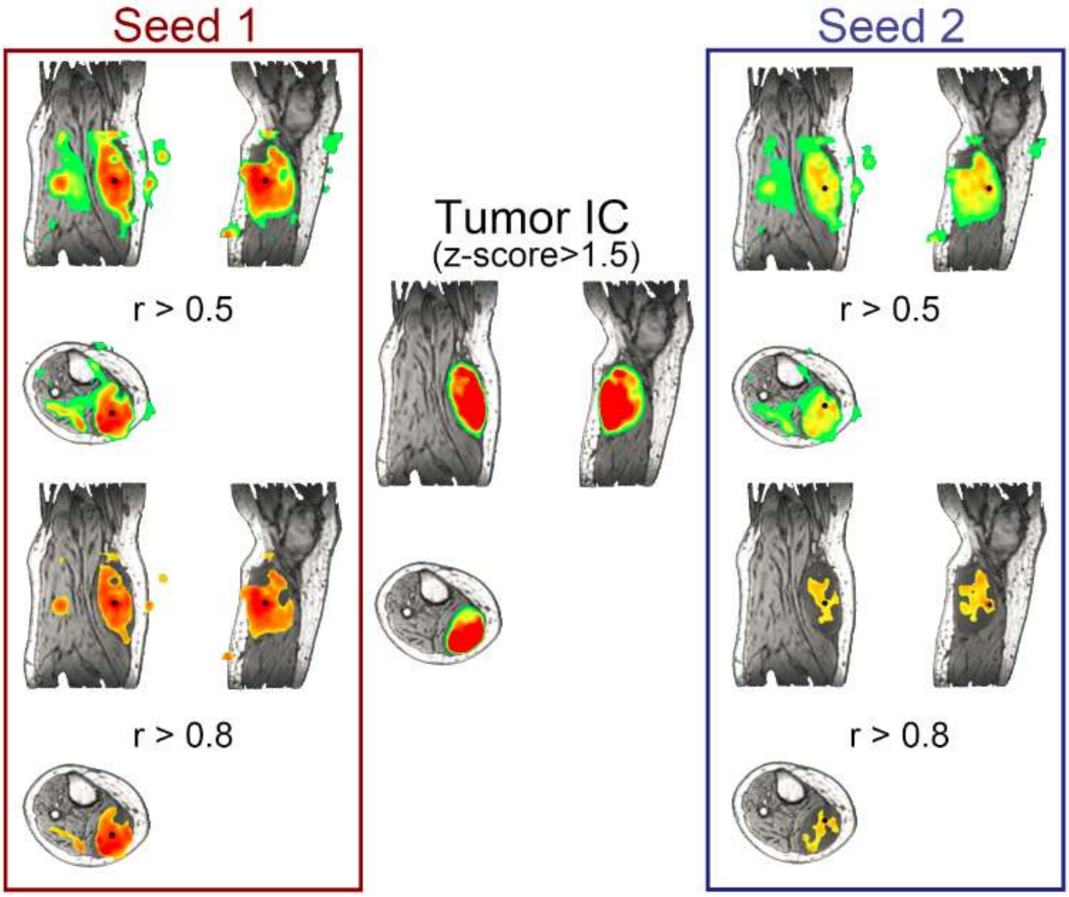

Supplementary Figure S4. Representative case (P #8 from Center 4) showing the spatial maps of the non-brain MSK tumor-related component derived from ICA on rs-fMRI and identified by DICI (Tumor IC) as well as those derived by seed-based correlations using two different seeds (Seed 1 and Seed 2) and different thresholds (0.5 and 0.8).

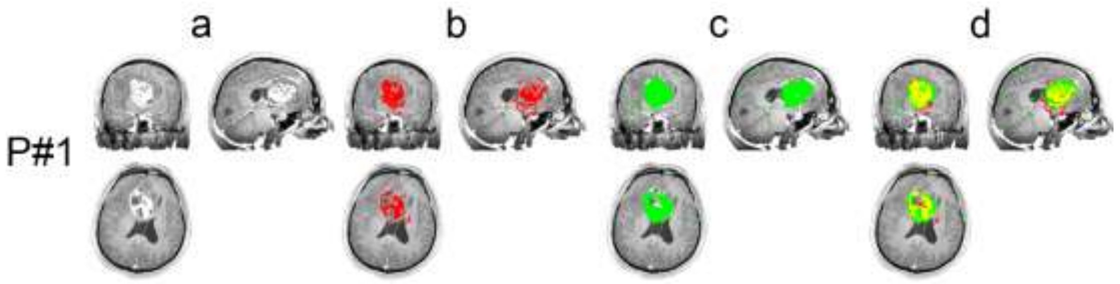

Supplementary Figure S5. Comparison between T1-based and rs-fMRI-based tumor segmentation. P #1 with high-grade glioma in the medial frontal area was randomly selected as an example. (a) Contrast-enhanced T1 image; (b) Tumor tissue (in red) delineated by using an intensity-based method based on the contrast-enhanced regions in (a); (c) Tumor-related component (in green) identified by using our method based on BOLD rs-fMRI data of this patient; (d) The overlap and differences between the two results in (b) and (c), where yellow color indicates the overlapping regions.

Supplementary Table S1.

DICI report with IC label and DICI values for all TNCs settings (P #2 from Center 1).

TNCs	IC label-1st	DICI value	IC label-2nd	DICI value	IC label-3rd	DICI value	IC label-4th	DICI value	1st-2nd	2nd-3rd	3rd-4th
10	8	2.110	3	0.590	9	0.330	10	0.053	1.520	0.260	0.277
20	10	2.384	16	0.626	18	0.172	13	-0.050	1.758	0.454	0.221
30	16	2.387	12	0.383	27	0.319	30	0.243	2.004	0.064	0.076
40	24	2.196	25	0.921	7	0.532	22	0.395	1.275	0.389	0.137
50	39	2.235	42	1.159	35	1.009	47	0.792	1.076	0.150	0.217
60	56	2.492	33	0.967	40	0.717	27	0.587	1.525	0.251	0.130
70	60	1.461	54	1.005	52	0.853	58	0.816	0.456	0.152	0.038
80	69	1.428	36	0.993	77	0.752	47	0.746	0.435	0.241	0.006
90	56	1.2612	51	1.185	70	0.781	41	0.684	0.076	0.404	0.097
100	83	1.334	51	1.211	53	1.033	87	0.988	0.124	0.178	0.045

DICI: Discriminability Index-based Component Identification; IC: independent component; TNCs: total number of components; 1st denotes the largest DICI value; 2nd denotes the second largest DICI value; 3rd denotes the third largest DICI value; 4th denotes the fourth largest DICI value; 1st -2nd denotes the difference between the largest DICI value and the second largest DICI value. The others are defined by this analogy. Grey shade indicates the best tumor-related component as suggested by the largest DICI value (shown in red) across multiple TNCs.

Supplementary Table S2.
Demographic and pathological information of the patients scanned in Center 1.

Variables	Values
**Cases**	8
**Age (yrs)**	
Mean ± std	48±16
Range	14-68
**Males (females)**	3 (5)
**Pathology-no.**	
Oligodendroglioma	5
Astrocytoma	1
Glioblastoma	2

Supplementary Table S3.
Demographic and pathological information of the patients scanned in Center 2.

Variables	Values
**Cases**	4
**Age (yrs)**	
Mean ± std	27±10
Range	18-40
**Males (females)**	4 (0)
**Pathology-no.**	
Astrocytoma	2
N.A.[a]	2

[a] Suspected glioblastoma (WHO III-IV) based on T1 images (since they were not undergone craniotomy, their pathological information is not available).

Supplementary Table S4.
Demographic and pathological information of the patients scanned in Center 3[a].

Variables	Values
**Cases**	20
**Age (yrs)**	
Mean ± std	49±17
Range	16-70
**Males (females)**	13 (7)
**Pathology-no.**	
Glioblastoma	20

[a] Patients had glioblastomas (WHO IV), and they were randomly selected from a large database of Center 3.

Supplementary Table S5.
Description of the scanner information and acquisition parameters used in each center.

Center	Scanner (Field strength)	RF coil type	Echo Planar Imaging	3D T1 Imaging
Center 1	GE DISCOVERY MR750 (3.0 T)	8-channel head coil	TR/TE = 2000/30 ms<br>Flip angle = 90°<br>Slices # = 43<br>Thickness = 3.2 mm<br>FOV = 220 mm×220 mm<br>Matrix = 64×64<br>Scanning time = 8 min	TR/TE = 8.156/3.18 ms<br>Flip angle = 8°<br>Slices # =176<br>Thickness = 1 mm<br>FOV = 256 mm×256 mm<br>Matrix = 256×256
Center 2	SIEMENS Verio3.0 (3.0 T)	8-channel head coil	TR/TE = 2000/30 ms<br>Flip angle = 90°<br>Slices # = 31<br>Thickness = 3 mm<br>FOV = 256 mm×256 mm<br>Matrix = 64×64<br>Scanning time = 8 min	TR/TE = 2000/2.98 ms<br>Flip angle = 9°<br>Slices # = 176<br>Thickness = 1 mm<br>FOV = 256 mm×256 mm<br>Matrix = 256×223
Center 3	SIEMENS MAGNETOM Verio (3.0 T)	8-channel head coil	TR/TE = 2000/35 ms<br>Flip angle = 90°<br>Slices # = 33<br>Thickness = 4 mm<br>FOV = 210 mm×210 mm<br>Matrix = 64×64<br>Scanning time = 8 min	TR/TE = 1900/2.93 ms<br>Flip angle = 9°<br>Slices # = 176<br>Thickness = 1 mm<br>FOV = 256 mm×256 mm<br>Matrix = 256×215
Center 4	SIEMENS Avanto (1.5 T)	8-channel knee coil<br>8-channel body array<br>a large circularly polarized flexible coil	TR/TE = 2000/40 ms<br>Flip angle = 90°<br>Slices # = 20<br>Thickness = 5 mm<br>FOV = 220 mm×220 mm<br>Matrix = 64×64<br>Scanning time = 6 min	TR/TE = 1900/2.97 ms<br>Flip angle = 15°<br>Slices # = 176<br>Thickness = 1 mm<br>FOV = 220 mm×220 mm<br>Matrix = 256×246

TR: repetition time; TE: echo time; slices #: slices number; FOV: field of view; RF: Radio Frequency

Supplementary Table S6.
Demographic and clinical information of the MSK tumor patients scanned in Center 4[a].

Variables	Values
**Cases**	28
**Age (yrs)**	
Mean ± std	38 ± 19
Range	11-73
**Males (females)**	19 (9)
**Position-no.**	
Femur	12
Tibia	4
Fibula	2
Humerus	1
Calcaneus	1
Pelma	1
Ilium	1
Soft tissue	6

[a] Patients had malignant musculoskeletal (MSK) tumors, and they were randomly selected from a large database of Center 4.

Supplementary Table S7.

DICI report with IC label and DICI values for all TNCs settings (P #2 from Center 1 with $z > 0.5$).

TNCs	IC label-1st	DICI value	IC label-2nd	DICI value	IC label-3rd	DICI value	IC label-4th	DICI value	1st-2nd	2nd-3rd	3rd-4th
10	8	1.488	3	0.801	9	0.312	10	0.092	0.687	0.489	0.220
20	10	1.586	16	0.595	14	0.245	2	0.211	0.992	0.350	0.034
30	16	1.682	27	0.387	3	0.334	20	0.226	1.296	0.053	0.108
40	24	1.618	25	0.809	31	0.518	35	0.412	0.809	0.291	0.106
50	39	1.599	42	0.982	35	0.850	36	0.670	0.617	0.133	0.180
60	56	1.905	33	0.749	40	0.556	19	0.496	1.156	0.193	0.060
70	60	1.257	54	0.847	58	0.710	43	0.704	0.411	0.137	0.006
80	69	1.261	36	0.915	77	0.716	61	0.658	0.346	0.200	0.058
90	56	1.011	51	1.007	70	0.691	54	0.504	0.004	0.316	0.187
100	83	1.182	51	1.006	53	0.762	87	0.723	0.176	0.244	0.039

DICI: Discriminability Index-based Component Identification; IC: independent component; TNCs: total number of components; 1st denotes the largest DICI value; 2nd denotes the second largest DICI value; 3rd denotes the third largest DICI value; 4th denotes the fourth largest DICI value; 1st -2nd denotes the difference between the largest DICI value and the second largest DICI value. The others are defined by this analogy. Grey shade indicates the best tumor-related component as suggested by the largest DICI value (shown in red) across multiple TNCs.

Supplementary Table S8.

DICI report with IC label and DICI values for all TNCs settings (P #2 from Center 1 with $z > 1.5$).

TNCs	IC label-1st	DICI value	IC label-2nd	DICI value	IC label-3rd	DICI value	IC label-4th	DICI value	1st-2nd	2nd-3rd	3rd-4th
10	8	2.446	9	0.409	3	0.150	5	0.073	2.037	0.259	0.077
20	10	2.902	16	0.555	18	0.274	11	-0.082	2.347	0.281	0.357
30	16	2.958	12	0.535	30	0.494	20	0.171	2.423	0.041	0.323
40	24	2.609	25	0.942	7	0.663	35	0.435	1.667	0.279	0.228
50	39	2.747	42	1.175	35	1.030	47	0.850	1.572	0.145	0.181
60	56	2.963	33	1.042	40	0.767	36	0.746	1.921	0.276	0.021
70	60	1.710	54	1.115	52	0.906	43	0.884	0.595	0.209	0.022
80	69	1.769	36	1.065	77	0.992	46	0.903	0.704	0.073	0.089
90	56	1.590	51	1.328	70	0.873	41	0.734	0.262	0.455	0.139
100	83	1.520	53	1.457	51	1.368	87	1.310	0.062	0.090	0.057

DICI: Discriminability Index-based Component Identification; IC: independent component; TNCs: total number of components; 1st denotes the largest DICI value; 2nd denotes the second largest DICI value; 3rd denotes the third largest DICI value; 4th denotes the fourth largest DICI value; 1st -2nd denotes the difference between the largest DICI value and the second largest DICI value. The others are defined by this analogy. Grey shade indicates the best tumor-related component as suggested by the largest DICI value (shown in red) across multiple TNCs.

Supplementary Table S9.

DICI report with IC label and DICI values for all TNCs settings (P #2 from Center 1 with $z > 2$).

TNCs	IC label-1st	DICI value	IC label-2nd	DICI value	IC label-3rd	DICI value	IC label-4th	DICI value	1st-2nd	2nd-3rd	3r[illegible]
10	8	2.139	9	0.459	5	0.143	2	-0.359	1.680	0.316	[illegible]
20	10	2.935	16	0.359	18	0.335	13	-0.248	2.576	0.024	[illegible]
30	16	3.090	12	0.610	30	0.089	19	-0.143	2.480	0.521	[illegible]
40	24	2.741	25	0.876	7	0.726	39	0.230	1.865	0.150	[illegible]
50	39	2.774	42	1.139	35	0.958	6	0.765	1.635	0.181	[illegible]
60	56	3.117	33	0.918	36	0.853	27	0.784	2.198	0.065	[illegible]
70	60	1.918	54	1.047	58	0.987	43	0.928	0.871	0.060	[illegible]
80	69	1.998	46	1.256	77	1.152	36	1.121	0.742	0.104	[illegible]
90	56	1.875	51	1.378	70	0.987	27	0.729	0.496	0.392	[illegible]
100	83	1.827	53	1.821	87	1.460	51	1.422	0.007	0.361	[illegible]

DICI: Discriminability Index-based Component Identification; IC: independent component; TNCs: total number of components; 1st denotes the largest DICI value; 2nd denotes the second largest DICI value; 3rd denotes the third largest DICI value; 4th denotes the fourth largest DICI value; 1st -2nd denotes the difference between the largest DICI value and the second largest DICI value. The others are defined by this analogy. Grey shade indicates the best tumor-related component as suggested by the largest DICI value (shown in red) across multiple TNCs.

Supplementary Table S10.

DICI report with IC label and DICI values for all TNCs settings (P #2 from Center 1 with $z > 2.5$).

TNCs	IC label-1st	DICI value	IC label-2nd	DICI value	IC label-3rd	DICI value	IC label-4th	DICI value	1st-2nd	2nd-3rd	3rd-4th
10	9	0.310	5	0.119	2	-0.363	1	Inf	0.191	0.482	Inf
20	10	2.820	18	0.395	16	0.393	13	-0.236	2.424	0.002	0.630
30	16	2.756	12	0.656	19	-0.136	29	-0.505	2.099	0.793	0.369
40	24	2.782	25	0.823	7	0.796	39	0.389	1.959	0.027	0.406
50	39	2.829	42	1.017	35	0.908	6	0.825	0.812	0.109	0.083
60	56	2.837	36	0.927	27	0.821	33	0.806	1.910	0.106	0.015
70	60	1.929	58	1.182	54	0.960	43	0.837	0.747	0.222	0.123
80	46	NaN	69	2.101	77	1.220	61	1.191	NaN	0.882	0.028
90	56	1.935	51	1.476	70	1.111	49	0.750	0.460	0.365	0.361
100	53	1.991	83	1.792	87	1.522	51	1.511	0.199	0.270	0.010

DICI: Discriminability Index-based Component Identification; IC: independent component; TNCs: total number of components; 1st denotes the largest DICI value; 2nd denotes the second largest DICI value; 3rd denotes the third largest DICI value; 4th denotes the fourth largest DICI value; 1st -2nd denotes the difference between the largest DICI value and the second largest DICI value. The others are defined by this analogy. Grey shade indicates the best tumor-related component as suggested by the largest DICI value (shown in red) across multiple TNCs. NaN means there was no voxels survived after applying the threshold.

Supplementary Table S11.

DICI report with IC label and DICI values for all TNCs settings (P #2 from Center 1 with $z > 3$).

TNCs	IC label-1st	DICI value	IC label-2nd	DICI value	IC label-3rd	DICI value	IC label-4th	DICI value	1st-2nd	2nd-3rd	3rd-4th
10	3	NaN	8	NaN	5	0.162	9	0.014	NaN	NaN	0.148
20	10	NaN	18	0.390	13	-0.180	9	-0.452	NaN	0.571	0.272
30	16	NaN	12	0.765	19	-0.213	29	-0.561	NaN	0.978	0.349
40	24	NaN	7	0.795	25	0.703	39	0.450	NaN	0.092	0.253
50	39	NaN	42	0.933	35	0.861	6	0.848	NaN	0.071	0.013
60	56	2.636	36	0.932	27	0.787	33	0.612	1.704	0.145	0.175
70	60	1.998	58	1.377	54	0.876	51	0.792	0.621	0.501	0.084
80	46	NaN	49	NaN	69	2.081	61	1.310	NaN	NaN	0.771
90	56	1.963	51	1.407	70	1.135	27	0.736	0.556	0.272	0.399
100	53	2.045	83	1.796	87	1.512	51	1.449	0.249	0.285	0.063

DICI: Discriminability Index-based Component Identification; IC: independent component; TNCs: total number of components; 1st denotes the largest DICI value; 2nd denotes the second largest DICI value; 3rd denotes the third largest DICI value; 4th denotes the fourth largest DICI value; 1st -2nd denotes the difference between the largest DICI value and the second largest DICI value. The others are defined by this analogy. Grey shade indicates the best tumor-related component as suggested by the largest DICI value (shown in red) across multiple TNCs. NaN means there was no voxels survived after applying the threshold.
